# Perinatal maternal characteristics predict a high risk of neonatal asphyxia: A multi-center retrospective cohort study in China

**DOI:** 10.3389/fmed.2022.944272

**Published:** 2022-08-08

**Authors:** Yi Yu, Jinsong Gao, Juntao Liu, Yabing Tang, Mei Zhong, Jing He, Shixiu Liao, Xietong Wang, Xinghui Liu, Yinli Cao, Caixia Liu, Jingxia Sun

**Affiliations:** ^1^Department of Obstetrics and Gynecology, Peking Union Medical College Hospital, Beijing, China; ^2^Department of Obstetrics and Gynecology, National Clinical Research Center for Obstetrics & Gynecologic Diseases, Peking Union Medical College Hospital (CAMS), Beijing, China; ^3^Department of Obstetrics and Gynecology, Hunan Maternal and Child Health Care Hospital, Changsha, China; ^4^Department of Obstetrics and Gynecology, Nanfang Hospital Southern Medical University, Guangzhou, China; ^5^Department of Obstetrics and Gynecology, Women's Hospital School of Medicine Zhejiang University, Hangzhou, China; ^6^Department of Obstetrics and Gynecology, Henan Provincial People's Hospital Zhengzhou, Henan, China; ^7^Department of Obstetrics and Gynecology, Shandong Provincial Hospital Affiliated to Shandong University, Jinan, China; ^8^Department of Obstetrics and Gynecology, Sichuan University West China Second Hospital, Chengdu, China; ^9^Department of Obstetrics and Gynecology, Northwest Women and Children's Hospital, Xi'an, China; ^10^Department of Obstetrics and Gynecology, Shengjing Hospital Affiliated to China Medical University, Shenyang, China; ^11^Department of Obstetrics and Gynecology, The First Clinical Hospital Affiliated to Harbin Medical University, Harbin, China

**Keywords:** predictive model, neonatal asphyxia, risk factors, fetal distress, cohort study

## Abstract

**Objective:**

This study aimed to identify various perinatal maternal characteristics that contributed to neonatal asphyxia (NA) in term and late-preterm newborns based on the data obtained from a Chinese birth registry cohort and to establish an effective model for predicting a high risk of asphyxia.

**Method:**

We retrospectively reviewed and analyzed the birth database from July 1, 2016, to June 30, 2017, in the main economically developed regions of China. Asphyxia was defined as an Apgar score <7 at 5 min post-delivery with umbilical cord arterial blood pH < 7.2 in the infant born after 34weeks. We compared the perinatal maternal characteristics of the newborns who developed asphyxia (NA group, *n* = 1,152) and those who did not (no NA group, *n* = 86,393). Candidate predictors of NA were analyzed using multivariable logistic regression. Subsequently, a prediction model was developed and validated by an independent test group.

**Result:**

Of the maternal characteristics, duration of PROM ≥ 48 h, a gestational week at birth <37, prolonged duration of labor, hypertensive disorder, nuchal cord, and birth weight <2,500 or ≥4,000 g, abnormal fetal heart rate, meconium-stained amniotic fluid, and placenta previa were included in the predicting model, which presented a good performance in external validation (*c*-statistic of 0.731).

**Conclusion:**

Our model relied heavily on clinical predictors that may be determined before or during birth, and pregnant women at high risk of NA might be recognized earlier in pregnancy and childbirth using this methodology, allowing them to avoid being neglected and delayed. Future studies should be conducted to assess its usefulness.

## Introduction

Globally, the occurrence of neonatal asphyxia (NA) is between 0.5 and 1.0% in full-term neonates, which is higher in preterm newborns (1), and NA contributes to 23% of the main causes of neonatal death (2). NA is defined as the failure of neonates to initiate and sustain breathing at birth (3), followed by impaired gas exchange leading to progressive hypoxemia, hypercapnia, and significant metabolic acidosis if prolonged (4). Severe NA may cause multiple organ damage, including brain damage, cardiac injury, respiratory distress, renal injury, liver incompetence, and necrotizing enterocolitis, even endangers neonatal survival (4–10). Among these, brain damage, which is also termed hypoxic-ischemic encephalopathy (HIE), is of the greatest concern due to its high lethality and long-term neurological sequelae, like cerebral palsy, epilepsy, intellectual incompetence, cognitive deficits, and motor disability, thereby leaving the family and society a lifetime burden (11–13).

It remains a priority to identify the newborns that may experience NA and minimize the rate of NA. Socioeconomic factors have been demonstrated to be strongly related to NA, like low socioeconomic and educational status, inadequate antenatal care, and poor intrapartum care (14, 15). Moreover, medical factors like maternal obstetrical complications including pregnancy-induced hypertension disease and gestational diabetes, parity, early gestational age, low birth weight, premature rupture of membranes, a prolonged second stage of labor, shoulder dystocia, abnormal fetal heart rate (FHR), and intrauterine meconium staining have already been identified to be risk factors of NA (16–20).

To summarize, several antenatal factors and intrapartum events have been associated with the presence of NA. Nevertheless, it is difficult to identify fetuses at high risk of asphyxia and to classify them into appropriate monitoring and management strategies during prenatal and intrapartum care. This study aimed to develop and evaluate prognosis models for predicting NA by combining multiple predictors, identify women at risk of adverse birth outcomes, and improve maternal and neonatal management.

## Materials and methods

### Data sources and study design

This retrospective multi-center childbirth registration research analyzed birth data and delivery problems from 14 representative medical institutions (including two secondary and 12 tertiary hospitals) across 10 provinces in China's four major economic regions from October 1, 2016, to September 30, 2017. Clinicians at each hospital gathered and reported complete medical information for each birth into a pre-designed standardized data collecting system that relied on digital and written medical records. The study was authorized by the study centers' Institutional Ethics Committees. All patients consented to and signed a consent form for the gathering of data from their medical records, as well as the publishing of their medical information, at the time of registration.

**Figure 1:**
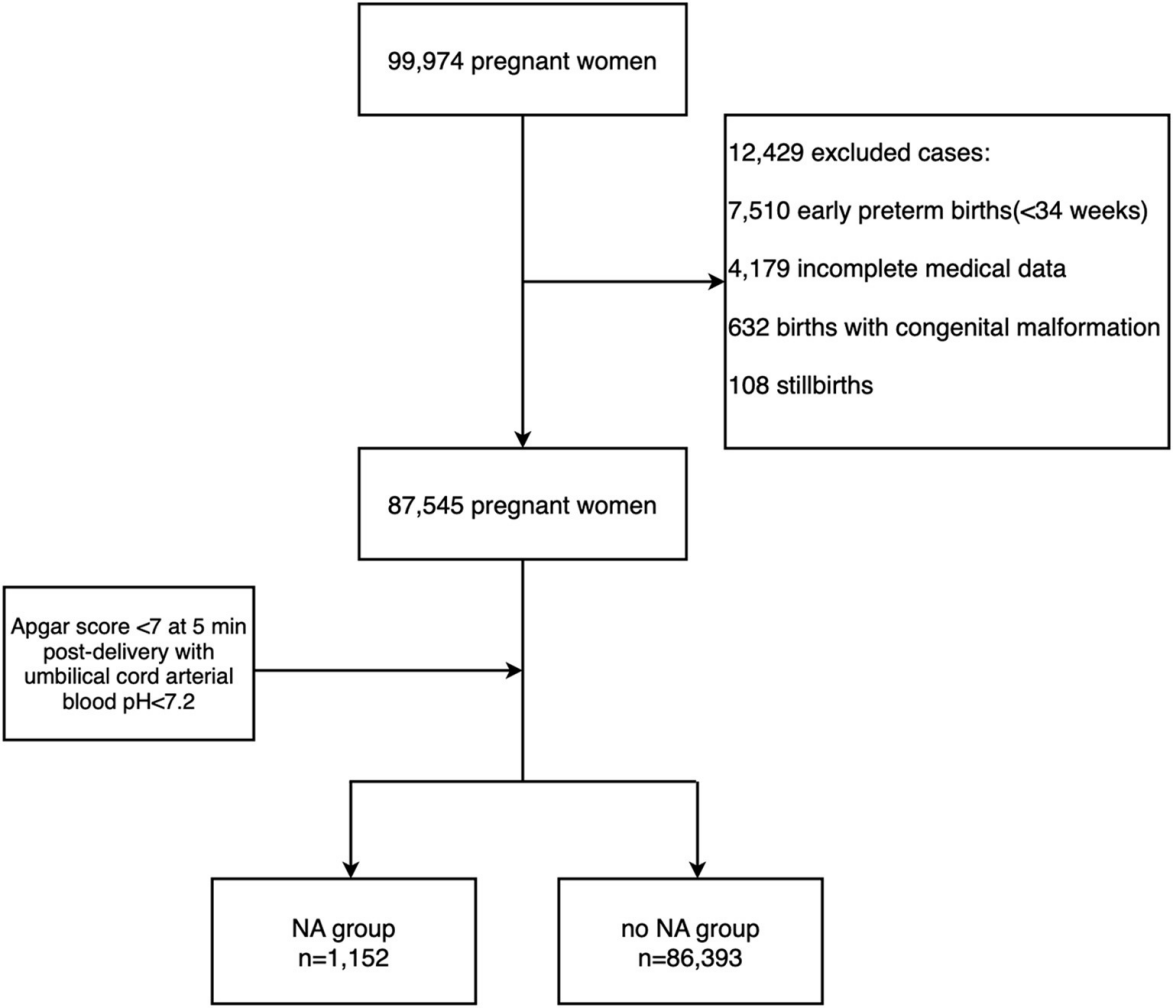
Flowchart of the included patients.

Since it is not recommended to establish the diagnosis of asphyxia by using the Apgar score alone (21, 22), the diagnosis for birth asphyxia was based on the committee opinion of the American College of Obstetrics and Gynecology (ACOG) (23) and World Health Organization (WHO): neonates in the term births and late-preterm births (24) with Apgar score <7 at 5 min post-delivery with umbilical cord arterial blood pH < 7.2 were diagnosed NA (25). Exclusion criteria included early-preterm newborns, stillbirth, congenital malformation, and incomplete medical data. Hence from the data of 99,974 pregnant women, a total of 87,545 pregnancies were selected for evaluation, including 1,152 pregnancies diagnosed with NA and 86,393 pregnancies that didn't complicate with NA. They were with complete basic information and with gestational week ≥34 weeks (Figure 1). In this case, we excluded the early-preterm newborns <34 gestational weeks.

The independent variables included in this study were: (1) maternal basic characteristics and antepartum-related factors: age, medical history, body mass index at birth, weight gain during pregnancy, the way of conception, gravidity and parity, and obstetric complication during pregnancy, (2) maternal intrapartum related factors: type of labor, mode of delivery, augmentation of labor, time of membrane rupture, duration of the second stage, the color of amniotic fluid, (3) neonatal related factors: fetal distress, birth weight, gestational age at birth and nuchal cord. Detailed information on the definition of the variables were shown in Table 1.

**Table 1:** Definition of variables included in study.

**Variables**	**Definition**
Parity	Number of previous pregnancies >28 weeks gestational age
Duration of second stage of labor	The time from when the cervix was fully dilated to the time of labor
Premature rupture of membranes (PROM)	Rupture of membranes before the onset of labor
Duration of PROM	Duration from rupture of membranes to labor
Fetal distress
Abnormal FHR with EFM	Non-reassuring Category II FHR tracings or Category III FHR according ACOG Committee on Practice Bulletins
Meconium stained amniotic fluid	Yellow or green coloration of amniotic fluid
Obstetric events
Hypertensive disorders	Blood pressure of 140/90 mmHg and above measured in prenatal care visits, including hypertension/(pre) eclampsia
Gestational diabetes mellitus	75-g oral glucose tolerance test (OGTT) at 24–28 weeks, when any one of the following plasma glucose values are met or exceeded fasting 5.1 mmol/L, 1 h 10.0 mmol/L, 2 h 8.5 mmol/L
Placenta previa	Placenta is covering the cervical confirmed by ultrasound after >32 weeks gestation
Placental abruption	Premature separation of the placenta before neonates was born
Uterine rupture	A defect that involves the entire uterine wall
Cord prolapse	Descent of the cord through the cervix, passing the presenting part
Nuchal cord	Umbilical cord coiled the fetus confirmed after birth
Abnormal placental morphology	Velamentous placenta, battledore placenta, lobulated placenta
Gestational week at birth	Estimated by last menstrual period or ultrasound
Postpartum hemorrhage	Blood loss ≥500 ml in vaginal delivery or ≥1,000 ml in cesarean section within 24 h after delivery

### Statistical analysis

Variables were compared between two outcomes (NA and no NA) using Python 3.8.5. Normal distributions were tested for all variables. The Student's *t*-test or Mann-Whitney U rank test was used to compare the numerical variables according to their distribution and the results are shown as means ± standard deviation (range) or as medians and interquartile ranges depending on their distribution. The chi-squared test was used to analyze categorical variables and the results are presented as percentages. Bonferroni-corrected *P*-values were calculated, and the Bonferroni-corrected alpha was set at 0.05. Variables having corrected *P* values < 0.05 were included in univariate analysis and multivariate logistic regression. Maternal age was also categorized into <35 and ≥35 y according to the cut-off value of advanced maternal age (26); duration of the second stage of labor was categorized into <2 and ≥2 h according to the definition of the prolonged second stage of labor (27); gestational week at birth and birth weight were categorized according to the definition of preterm/post-term birth and low/high birth weight (24, 28).

To contemplates the relationships that different variables have with each other, we performed Bartlett's test and the Kaiser-Meyer-Olkin (KMO) test to measure the suitability of data for factor analysis. In Bartlett's test, the *P*-value was 0, which was statistically significant and indicated that the observed correlation matrix was not an identity matrix. KMO test presented with a result of 0.55, which was not perfect for factorial analysis but would consider applicable. Then we calculated the response value and performed an exploratory factorial analysis by including all the significant variables.

At a 3:1 allocation ratio, a total of 87,545 patients were randomly divided into training and test groups. We verified the statistical significance of combinations of factors while choosing variables for the models. The training group was used to calculate the statistical significance of combinations of variables using univariate and multivariate logistic regression models. We estimated odds ratios (OR) and adjusted ORs with 95 percent confidence intervals (CI) and *P* values. As a result, the scoring system is formed.

The chi-square values from the logistic regression and *C*-statistics utilizing the test group from the receiver operating characteristic (ROC) analysis with sensitivity and specificity were used to confirm the scoring system's discriminating capacity. The prediction performance of the identified risk variables and scoring system was assessed using the area under the ROC curve (AUC). Statistical significance was defined as a *P* value of <0.05.

## Results

### Maternal and prenatal events related factors

Of the retrospective cohort consisting of 87,545 pregnancies, 1,152(1.3%) births complicated with NA were identified. The comparisons of the maternal characteristics of the NA group and the no NA group is presented in Table 2. The mean age of the pregnant women with and without NA were 30.05 ± 4.94 and 30.72 ± 4.5 years, respectively, which showed no differences between the two groups (*P* = 0.628), as well as the proportion of advanced maternal age. Among study participants, 733(63.6%) of the mothers with NA and 51,775(59.9%) of the mothers without NA were primiparous. There was a significant difference in parities of mothers between the two groups (*P* = 0.003). In addition, the body mass index at birth, weight gain during pregnancy, and the rate of assisted reproductive technology did not differ between the two groups, and the proportion of multiple births was also similar (*P* = 0.08).

**Table 2:** Maternal characteristics and perinatal events established for neonatal asphyxia [Data was given as mean ± SD or *n* (%)].

**Variable**	**Neonatal asphyxia**	**No neonatal asphyxia**	*p* value*
	***n* = 1,152**	***n* = 86,393**	
Maternal age, y***	30.05 ± 4.94	30.72 ± 4.5	
<35	934(81.1)	68,839(79.7)	0.628
≥35	218(18.9)	17,554(20.3)	
Parity	0.38 ± 0.52	0.42 ± 0.52	
Primiparous	733(63.6)	51,775(59.9)	0.0125
Multiparous	419(36.4)	34,618(40.1)	
BMI at birth, kg/m^2^	27.14 ± 4.04	26.80 ± 8.09	0.162
Weight-gain	15.58 ± 5.45	15.57 ± 5.25	0.937
ART	42(3.6)	2,758(3.1)	0.459
Fetal numbers			
Singleton	1,111(96.4)	84,055(97.3)	0.08
Multiple	41(3.6)	2,338(2.7)	
Medical history			
Diabetes mellitus	7(0.5)	412(0.5)	0.521
Nephropathy	5(0.4)	212(0.2)	<0.001
Anemia	114(9.9)	8,435(9.8)	0.855
Cardiopathy	21(1.8)	654(0.7)	<0.001
Hepatopathy	68(5.9)	3,433(3.9)	<0.001
Immune Disease	6(0.5)	145(0.1)	0.0127
Mode of delivery			
Vaginal delivery	537(46.6)	46,194(53.4)	<0.001
Vaginal delivery with instruments	96(17.9)	1,490(3.2)	<0.001
Cesarean section	615(53.4)	40,199(46.6)	<0.001
Emergent cesarean section	155(25.2)	4,543(11.3)	<0.001
Type of labor			
Spontaneous	1,071(93.0)	77,084(89.2)	0.258
Induced/Augmented	81(7.0)	9,309(10.8)	
Duration of second stage of labor, h***	1.08 ± 0.92	0.76 ± 12.9	
0–2	445(82.9)	39,102(84.6)	<0.001
≥2	92(17.1)	7,092(15.4)	
Premature rupture of membranes (PROM)	210(18.2)	12,338(14.2)	<0.001
Duration of PROM**	20.20 ± 24.01	18.01 ± 33.19	0.147
Fetal distress			
Abnormal FHR with EFM	147(12.7)	2,416(2.7)	<0.001
Meconium stained amniotic fluid	134(11.5)	3,364(3.9)	<0.001
Obstetric events			
Hypertensive disorders	122(11.6)	3,459(4.0)	<0.001
Gestational diabetes mellitus	150(13.0)	10,843(12.5)	0.445
Placenta previa	77(6.6)	2,100(2.4)	<0.001
Placental abruption	34(2.9)	425(0.4)	<0.001
Uterine rupture	5(0.4)	126(0.1)	0.039
Cord prolapsed	8(0.6)	59(0.07)	<0.001
Nuchal cord	380(32.9)	19,834(22.9)	<0.001
Abnormal placental morphology	141(12.2)	5,817(6.7)	<0.001
Gestational week at birth, wk***	38.30 ± 2.06	39.06 ± 1.41	<0.001
34–37	299(26.0)	6,208(7.2)	
37–41	852(74.0)	80,071(92.7)	
≥42	1(0.08)	114(0.13)	
Fetal gender			0.042
Male	637(55.3)	44,751(51.8)	
Female	515(44.7)	41,642(48.2)	
Birth weight, g***	3078.92 ± 662.79	3300.27 ± 463.90	
<2,500	229(19.9)	4,320(5.1)	<0.001
2,500–4,000	850(73.8)	77,321(89.5)	
≥4,000	73(6.3)	4,752(5.4)	
Postpartum hemorrhage	94(8.1)	4,374(5.0)	<0.001
NICU admission	949(82.4)	10,786(12.4)	<0.001
Resuscitation of newborn	446(38.7)	276(0.3)	<0.001
Immediate endotracheal intubation	89(7.7)	27(0.03)	<0.001

^*^Bonferroni-corrected alpha was set at 0.05. Variables having Bonferroni-corrected P-values < 0.05 were significant.

^**^Mann-Whitney U rank tests were used.

^***^Student's t-test were used on numeric forms and Chi-square test were used on categorical forms.

Pre-pregnancy complications are shown in Table 2. The mothers complicated with heart disease, kidney disease, liver complications, and immune disease before getting pregnant were predisposed to arise NA. However, the occurrences of pre-existing diabetes mellitus and anemia weren't significantly higher in the NA group. Perinatal obstetric complications are also presented in Table 2. Mothers complicated with hypertensive disorders (11.6%NA vs. 2.7% no NA, *P* < 0.001), placenta previa (6.6%NA vs. 2.4% no NA, *P* < 0.001), placental abruption (2.9%NA vs. 0.4% no NA, *P* < 0.001), uterine rupture (0.4%NA vs. 0.1% no NA, *P* < 0.001), cord prolapse (11.6%NA vs. 2.7% no NA, *P* < 0.001), nuchal cord (11.6%NA vs. 2.7% no NA, *P* = 0.039), and abnormal placental morphology (12.2%NA vs. 6.7% no NA, *P* < 0.001) were more likely to develop NA. Surprisingly, the presences of gestational diabetes mellitus were similar in the two groups (13.0%NA vs. 12.5% no NA, *P* = 0.445).

The maternal intrapartum characteristics are also shown in Table 2. Labor started spontaneously in 1,071(93.0%) of the NA group compared with 7,084(89.2%) no NA group (*P* = 0.258). The median duration of the second stage of labor was 0.77 ± 0.92 in the NA group and was 0.55 ± 12.9 in the group without NA, which were statistically different among the two groups (*P* < 0.001). Premature rupture of membranes (PROM) was presented in 210(18.2%) of the NA compared to 12,338(14.2%) of the no NA group (*P* < 0.001), but the duration of PROM showed no differences (*P* = 0.147). Of the enrolled pregnant women, cesarean sections were performed in 615(53.4%) patients from the NA group as compared to 40,199(46.6%) of the no NA group, the rate was significantly higher in the NA group (*P* < 0.001). The proportion of vaginal delivery with instruments among vaginal delivery and emergent cesarean section among cesarean section were 17.9 and 25.2% in the NA group, respectively, the rates of which were significantly lower in the no NA group, only 3.2 and 11.3% of the patients accepted instrumented vaginal delivery and emergent cesarean section, respectively (*P* < 0.001). Consistent with previous research, the two groups had significant differences in the presence of fetal distress, including abnormal fetal heart rate (FHR) monitored by an electrical fetal monitor (EFM) device (12.7% NA vs. 2.7% no NA, *P* < 0.001) and meconium-stained amniotic fluid (11.5% NA vs. 3.9% no NA, *P* < 0.001), which were more commonly seen in mothers with NA.

As for the neonatal characteristics, Table 2 also showed the detailed results of the gestational week at birth and birth weight, of which the two groups were significantly different not only calculated as numerical variables but also when categorized into three groups (*P* < 0.001). The neonates complicated with asphyxia tended to be smaller in gestational week and birth weight. Interestingly, our study showed that male newborns seem to be more likely to develop NA (55.3% NA vs. 51.8% no NA, *P* = 0.042). Of the patients who had NA, 949(82.4%) of the neonates were admitted to NICU, and only 12.4% of neonates without NA were transferred to NICU (*P* < 0.001). Furthermore, the use of resuscitation of newborns (38.7% NA vs. 0.3% no NA, *P* < 0.001) and immediate endotracheal intubation (7.7% NA vs. 0.03% no NA, *P* < 0.001) were more common in the NA group. Of the maternal outcomes, the occurrence of postpartum bleeding was also significantly higher in the NA group (8.1% NA vs. 5.0% no NA, *P* < 0.001).

### Selection of variables for discriminating the high-risk group and scoring system

According to the exploratory factorial analysis, seven factors were used for hypothesis testing since their eigenvalues were over 1 (Table 3). The interactions were not important by analyzing the loadings within each factor, except for the factors 5 and 7. There was a connection between the nuchal cord and cesarean section within factor 5 and a connection between the gestational week at birth and birth weight within factor 7. However, the variables didn't cluster, so we limit ourselves to comparing the main effects within each factor.

**Table 3:** Matrix of load of factors.

	**1**	**2**	**3**	**4**	**5**	**6**	**7**
Multiparous	−0.0930	0.0627	−0.0030	**0.9713**	−0.0695	−0.1707	−0.1107
Hypertensive disorders	−0.0119	−0.0106	−0.0082	−0.0075	−0.0040	−0.0011	0.1730
Fetal distress	0.0284	0.0462	0.0517	0.0025	0.0688	**0.8654**	−0.0513
Nuchal cord	0.0023	0.0227	−0.0283	−0.0576	**0.6764**	−0.0332	−0.0854
Gestational week at birth	0.0709	0.0083	0.0231	−0.0121	0.0055	0.0110	**0.5518**
Premature rupture of membranes (PROM)	**0.9917**	−0.0071	−0.0180	−0.0647	0.0185	−0.0148	−0.1051
Abnormal placental morphology	0.0171	−0.0118	0.0442	−0.0048	0.3040	0.0057	0.0040
Cesarean section	−0.3813	−0.3814	−0.3698	−0.3265	**−0.6415**	−0.1805	−0.2594
Maternal age ≥ 40 y	−0.0080	−0.0012	−0.0114	0.1223	0.0043	−0.0109	0.0085
Duration of PROM ≥ 48 h	0.1716	0.0081	0.0146	−0.0193	0.0170	0.0111	0.0561
Birth weight < 2,500	0.0554	−0.0128	−0.0304	0.0143	−0.0273	−0.0093	**0.4967**
Birth weight > 4,000	−0.0042	0.0253	0.0257	0.0129	0.0213	0.0086	−0.0045
Vaginal delivery with instruments	0.0341	0.1332	0.0393	−0.0284	0.0643	0.1575	0.0088
Duration of second stage of labor ≥2 h	0.0115	**0.9922**	0.0277	0.0266	−0.0526	−0.0586	−0.0606
Induced/Augmented labor	0.0589	0.0481	**0.9802**	−0.1436	0.0776	−0.0578	−0.0468
Emergent cesarean section	−0.0141	−0.0473	−0.0499	−0.0859	−0.0565	0.2381	0.0148

Variables within each factor having loadings larger than 0.4 were bold.

Univariate and multivariate logistic regression analyses were performed in the training group to evaluate the risk factors associated with NA (Table 4). After adjustments for possible effects of confounding variables, age ≥40 y (aOR 1.159, 95% CI 0.977–1.375), nulliparity (aOR 1.138, 95% CI 1.059–1.221), vaginal delivery with a normal duration of the second stage of labor (aOR 1.077, 95% CI 1.001–1.159), PROM (aOR 1.196, 95% CI 0.761–1.315), and a gestational week at birth ≥42 wk (aOR 1.128, 95% CI 0.738–1.467) didn't increase the risk of NA significantly.

**Table 4:** Univariate and multivariate logistic regression to evaluate the impact of variables on the presence of neonatal asphyxia.

**Variables**	**OR**	**aOR**	**95%CI**	***p* value**
Age ≥ 40 y	1.29	1.159	0.977–1.375	0.09
Nulliparity	2.237	1.138	1.059–1.221	<0.001
Premature rupture of membranes (PROM)	1.383	1.196	0.761–1.315	<0.001
Duration of PROM ≥ 48 h	1.635	1.311	0.972–1.766	0.076
Vaginal delivery	1.344	1.077	1.001–1.159	0.048
Duration of second stage of labor ≥ 2 h	1.368	1.412	1.235–1.613	<0.001
Abnormal FHR with EFM	2.038	4.754	3.749–5.817	<0.001
Meconium stained amniotic fluid	1.847	3.891	2.447–5.593	<0.001
Hypertensive disorders	1.371	2.387	2.092–2.723	<0.001
Placenta previa	1.428	2.827	2.409–3.315	<0.001
Nuchal cord	1.429	1.682	1.564–1.809	<0.001
**Gestational week at birth, wk**
34–37	1.556	2.172	1.985–2.377	<0.001
≥42	1.308	1.128	0.738–1.467	<0.001
**Birth weight, g**
<2,500	4.868	3.005	2.672–3.380	<0.001
≥4,000	1.468	1.486	1.301–1.699	<0.001

To estimate a more precise model for predicting, we included eight variables with higher aOR to be determinants of NA and assigned scores based on aOR values of the factors. The cut-off value for a prolonged duration of the second stage and a prolonged time of membrane rupture was set at 2 h (27) and 48 h (29), respectively. A scoring system as shown in Table 5, duration of PROM ≥48 h (aOR 1.311, 95% CI 0.972–1.766), vaginal delivery with a prolonged duration of the second stage (aOR 1.412, 95% CI 1.235–1.613), nuchal cord (aOR 1.682, 95% CI 1.564–1.809), and birth weight ≥4,000 (aOR 1.486, 95% CI 1.301–1.699) were assigned one point. While hypertensive disorder (aOR 2.387, 95% CI 2.092–2.723), gestational week <37 wk (aOR 2.172, 95% CI 1.985–2.377), and placenta previa (aOR 2.827, 95% CI 2.409–3.315) were assigned two points. Abnormal FHR with EFM (aOR 4.754, 95% CI 3.749–5.817) or meconium-stained amniotic fluid (aOR 3.891, 95% CI 2.447–5.593), and birth weight <2,500 g (aOR 3.005, 95% CI 2.672–3.380) were assigned four points due to their high coefficient. Each patient was evaluated according to the scoring system.

**Table 5:** A clinical characteristics scoring system for predicting NA.

**Variables**	**0**	**1**	**2**	**4**
Premature rupture of membranes (PROM)	<48 h	≥48 h	/	/
Duration of second stage of labor	CS or <2 h	≥2 h	/	/
Fetal distress	No	/	/	Abnormal FHR with EFM or Meconium stained amniotic fluid
Hypertensive disorders	No	/	Yes	/
Placenta previa	No	/	Yes	/
Nuchal cord	No	Yes	/	/
Gestational week at birth, wk	≥37	/	<37	/
Birth weight, g	2,500–4,000	≥4,000	/	<2,500

**Figure 2:**
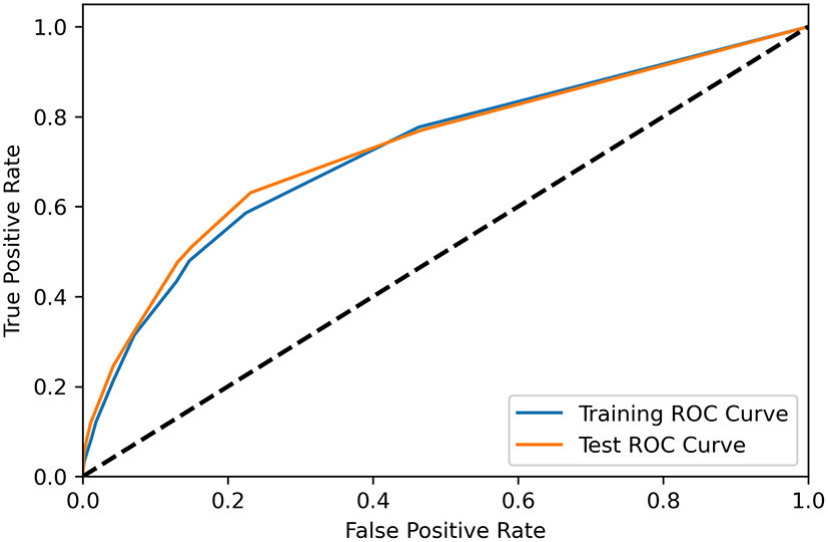
The receiver operating characteristic curve demonstrated the performance of the scoring system for predicting NA in the training group (blue) and test group (orange).

Table 6 showed the different cut-off score's sensitivity and specificity, in distinguishing patients from the NA group and no NA group. During the performance study, the scoring system performed best when the cut-off value was set at four points, with a specificity of 0.871, a sensitivity of 0.433, and an accuracy of 0.865 in predicting NA. The scoring system was validated to perform also well in the independent testing group, with a specificity of 0.869, a sensitivity of 0.476, and an accuracy of 0.863. The AUC of the training group and the test group were 0.724 and 0.731, respectively, as indicated in the ROC curve of the predictive model in Figure 2.

**Table 6:** Sensitivity, specificity and accuracy of different cut-off scores.

**Cut-off score**	**0**	**1**	**2**	**3**	**4**	**5**	**6**	**7**	**8**	**9**	**10**	**11**	**12**	**13**	**14**	**15**	**16**
Training group																
Sensitivity	1.000	0.776	0.586	0.479	0.433	0.314	0.213	0.120	0.086	0.042	0.032	0.016	0.009	0.003	0.000	0.000	0.000
Specificity	0.000	0.537	0.775	0.853	0.871	0.929	0.958	0.982	0.988	0.996	0.998	0.999	1.000	1.000	1.000	1.000	1.000
Accuracy	0.013	0.540	0.772	0.848	0.865	0.921	0.948	0.971	0.976	0.984	0.985	0.986	0.987	0.987	0.987	0.987	0.987
Test group																
Sensitivity	1.000	0.770	0.631	0.512	0.476	0.317	0.246	0.143	0.119	0.067	0.052	0.016	0.012	0.004	0.004	0.000	0.000
Specificity	0.000	0.533	0.769	0.849	0.869	0.931	0.958	0.983	0.989	0.997	0.998	0.999	1.000	1.000	1.000	1.000	1.000
Accuracy	0.015	0.537	0.766	0.844	0.863	0.922	0.947	0.970	0.976	0.982	0.984	0.985	0.985	0.984	0.985	0.985	0.985

Using exploratory data analysis, we analyzed the influence of the variables on the APGAR scores among the NA group, from both the average APGAR score and the rate of severe NA, defined by an APGAR score of <3 at 5 min after birth. As shown in Table 7, the variables of multiparous, hypertensive disorders, placenta previa, cesarean section, maternal age ≥40 y, duration of the second stage of labor ≥2 h, birth weight <2,500 g, and the gestational week at birth <37 weeks were associated with lower APGAR score and a higher rate of severe NA.

**Table 7:** Influence of the variables in the APGAR scores.

**Variables**	**Average**	**Rate of**
	**APGAR score**	**severe NA (%)**
Parity
Primiparous	6.11	6.0%
Multiparous	5.73	13.0%
Hypertensive disorders
Without	6.00	8.5%
With	5.73	12.7%
Fetal distress
Without	6.00	8.8%
With	5.88	9.5%
Nuchal cord
Without	5.88	10.2%
With	6.15	6.3%
Placenta previa
Without	5.99	8.8%
With	5.72	10.9%
Gestational diabetes mellitus
Without	5.97	8.9%
With	5.00	0.0%
Mode of delivery
Cesarean section	5.79	12.1%
Vaginal delivery	6.24	4.9%
Vaginal delivery with instruments	5.89	6.7%
Maternal age, y
<40	5.97	8.8%
≥40	6.04	10.6%
Duration of PROM, h
None	5.94	9.4%
0–48	6.08	7.6%
≥48	6.47	0.0%
Duration of second stage of labor, h
None	5.79	12.1%
0–2	6.23	4.8%
≥2	5.92	7.6%
Birth weight, g
<2,500	6.12	18.3%
2,500–4,000	5.47	6.3%
≥4,000	5.84	10.1%
Gestational week at birth, wk
34–37	5.57	15.7%
37–41	6.29	5.1%
≥42	5.97	5.9%

## Discussion

NA may lead to severe hypoxic-ischemic organ damage especially encephalopathy in newborns followed by severe long-term sequelae, even perinatal death. Hence, we conducted a nationwide multi-center retrospective cohort study of 87,545 pregnancies to develop a model for predicting NA in women who delivered in major economic regions of China. The overall prevalence of NA among newborns was 1.3% in these middle-high income regions. Compared with previous studies conducted in low- and middle-income countries, our database was collected from developed regions, where neonates are less likely to be born with NA (30, 31). A predictive model was developed by using both clinical prenatal, pre-delivery, and delivery variables in the training group and showed a good discriminative ability validated by the independent test group (*c*-statistic 0.731).

According to earlier research, nulliparity may be a risk factor for birth asphyxia and poor newborn outcomes (32, 33), though the association was not obvious (aOR 1.138, 95% CI 1.059–1.221) in our study. It is generally agreed that primiparous pregnant women had longer labor than multiparous pregnant women during vaginal birth (34). Hence parity and labor duration were mixed in contributing to the risk of NA. In low- and middle-income nations, prolonged duration of the second stage has been shown to be substantially linked to an increased risk of asphyxia (25, 35–38). Prolonged labor, especially in the second stage, may indicate that there exists an abnormal fetal position or cephalopelvic disproportion, which increases the chance of birth trauma. And the attempt to speed up delivery by using oxytocin may also cause fetal distress due to the stress of too many uterine contractions (39, 40). The forceps delivery or vacuum extraction were also predisposed to be applied when the labor fails to progress, which exerts pressure on the newborn's brain and increases the risk of NA (37, 41). Our study also revealed a significantly higher rate of cesarean sections in the NA group in our cohort, the percentage of emergent cesarean sections in cesarean sections was significantly higher in the NA group (25.2% vs. 11.3%). However, the multivariate logistic regression didn't show a significant relationship between the mode of delivery and the risk of NA (vaginal delivery, aOR 1.077, 95% CI 1.001–1.159). In previous studies, NA is more associated with birth *via* cesarean section, of which the explanations were the lack of squeezing to fetal lungs by the vaginal canal and less surfactant secreted to the alveolar surface during delivery (42). Though in a clinical scenario of a neonate born with NA caused by birth trauma, it is inevitable for obstetricians to reconsider that the outcome would have been different if a cesarean had been performed instead of vaginal delivery, or performed earlier. However, it is unrealistic and unnecessary to predict whether the fetus will be damaged by labor and performing the cesarean in advance. On the one hand, the newborn outcomes of cesareans performed for emergency reasons conducted over 30 min after the decision to operate weren't worse than those performed sooner (43). On the other hand, more cesareans in first labors to prevent NA may actually increase the rate of uterine rupture for the subsequent pregnancy, hence increasing the overall risk of NA (44).

Prolonged duration from PROM to labor was found to be positively linked with NA in our study, which has been confirmed by multiple previous studies (20, 45, 46). The umbilical cord is no longer surrounded by the amniotic fluid following a PROM and might get squeezed directly by the uterine contractions. Because the flow of oxygen-rich blood to the newborn is halted when the cord is compressed, the baby may suffer from birth asphyxia. Another explanation might be that without the protection of the membrane, external bacteria could easily be passed to the infant, potentially resulting in neonatal infection, followed by NA. Preeclampsia was proved to be strongly associated with an elevated risk of NA, as indicated in other studies (47–49). This impact is mediated by fetal feeding and oxygenation being reduced as a result of uteroplacental vascular insufficiency, as well as an increased chance of delivering preterm (48). According to our study, NA was linked to the nuchal cord, which was analogous to Ethiopia's research (35, 49). One reasonable explanation is that a tight nuchal cord constricts umbilical arteries, resulting in hypoxia.

In our study, low-birth-weight and preterm delivery neonates were shown to be at a higher risk of having NA compared with normal-birth-weight and full-term birth babies, which was consistent with earlier research (35, 37, 50). We didn't include the early-preterm newborns in analyses, because our initial data processing suggested that gestational week at birth was a strong self-predicator of NA, especially in newborns smaller than 34 weeks. Additionally, premature and low-birth-weight neonates may suffer from multiple organ immaturity and complications, especially in the respiratory system (51), which may obscure other underlying risk factors causing NA in statistical analyses. Post-term pregnancy (49) and macrosomia (52) were also demonstrated to be correlated to adverse neonatal outcomes due to placental insufficiency and increased risk of shoulder dystocia during labor, respectively. We identified a higher risk of NA in male newborns when evaluating the neonatal data. This finding was also reported by Sunny et al. (53). Studies have been conducted in China (54) and Israel (55, 56) to investigate the association between fetal gender and adverse pregnancy outcomes. Women carrying male fetuses were at increased risk for operative vaginal delivery, non-reassuring FHR, and lower Apgar scores. One cause may be those male infants are more likely to be macrosomia (57). More diggings on the underlying causes need to be performed.

As mentioned above, the risk factors were analyzed by factorial analysis but they didn't cluster well. It is hard to discriminate among different types of variables by analyzing the raw database. Clinically, some intrapartum events are direct causes of NA such as placental abruption, uterine rupture, cord prolapse, and severe shoulder dystocia, which might result in an abruptly disturbed blood supply to the fetus, since the placenta and umbilical cord are the key points of fetal feeding and oxygenation. However, most of these clinical episodes lack a sentinel event and are unexpected and unpredictable, necessitating urgent resuscitation (44). Nevertheless, these intrapartum events are related to some maternal comorbidities and complications. For instance, pregnancies complicated with placenta previa and hypertension disorder are at high risk of the presentation of placental abruption (58, 59), macrosomia is a risk factor for shoulder dystocia (60), and PROM may increase the potential of cord prolapse (61). So we included these “not so urgent” perinatal events in our scoring system to get a more advanced evaluation. When the fetal suffers from an oxygen deficit in the uterine, the first clinical presentation might be an abnormal FHR monitored by an electrical device or auscultation (62, 63). Though a marked increase in the cesarean delivery rate was related to the high false-positive rate of abnormal FHR (64), it was still well established that a non-reassuring category II is associated with low Apgar scores and neonatal intensive care unit (NICU) admission (65). Pruksanusak et al. (66) demonstrated a higher ability in predicting peripartum asphyxia of the combination of an abnormal five-tier FHR classification (67) and maternal-associated risk factors than the three-tier system (68). A longstanding non-reassuring fetal oxygen deficit may cause a release of intrauterine meconium, and the presence of meconium in the amniotic fluid increases the likelihood of meconium aspiration during intrauterine gasping or the first few breaths after delivery (69). In this case, the meconium amniotic fluid was widely identified as a risk factor for NA in former research (36, 38, 46, 49, 50, 70). We did not include vaginal delivery with instruments or emergent cesarean section in the scoring system because these two modes of labor may not be causal but merely associated with NA, which may be performed for a concerning fetal indication or obstetrical events that are themselves the key risk factor for NA, such as prolonged labor, non-reassuring FHR, meconium-stained amniotic fluid, placental and umbilical abnormalities.

Most previous studies were conducted in low- and middle-income countries. Our research was the first based on a database collected from the main economically developed regions of China as socioeconomic status plays an important role in the presence of NA. This is one of the few multi-center cohort studies focusing on predicting NA by including both characteristics during pre-delivery and delivery. Our model relied heavily on clinical predictors that may be determined before or during birth, such as a gestational week, fetal weight (which can be assessed before birth), hypertensive disorders, and the other risk factors described above. Compared with other predictive models from previous studies (37, 53, 70, 71), our predictive model is more clinically applicable, since the risk factors were quantified precisely by specifically assigned scores. And we accomplished a pretty high sensitivity by using as minimal variables as possible, making the model concise to the greatest extent possible. Our predictive scoring system could identify certain neonates that will experience NA prospectively, and could be the proper triaging of the obstetric risk assessment instrument. Pregnant women at high risk of NA might be recognized earlier in pregnancy and childbirth using this methodology, allowing them to avoid being neglected and delayed. Our study methods have several limitations. This was a retrospective cohort study, which limited the information of the generalized data. To be specific, the reasons for applying instruments during vaginal delivery or emergent cesarean section were not able to obtain from the raw data and the neonatal follow-ups, especially on the severity of HIE and other complications were absent. So this clinical score could not predict the severity of NA since it was not able to be quantified. This limitation can be resolved in a prospective study with a pre-designed protocol and detailed recording.

## Data availability statement

The original contributions presented in the study are included in the article/Supplementary material, further inquiries can be directed to the corresponding author/s.

## Ethics statement

The studies involving human participants were reviewed and approved by Peking Union Medical College Hospital Review Board (reference number: JS-1151). The patients/participants provided their written informed consent to participate in this study.

## Author contributions

YY wrote the manuscript and participated in data analysis. YT, MZ, JH, SL, XW, XL, YC, CL, and JS conducted data collection and quality control at their medical centers. JG conceived and designed the study. JL participated in designing the study and revising the language. All authors contributed to the article and approved the submitted version.

## Funding

This work was supported by the CAMS Innovation Fund for Medical Science (CIFMS) (2020-I2M-C&T-B-046) and Beijing Municipal Natural Science Foundation (No. 7212072).

## Conflict of interest

The authors declare that the research was conducted in the absence of any commercial or financial relationships that could be construed as a potential conflict of interest.

## Publisher's note

All claims expressed in this article are solely those of the authors and do not necessarily represent those of their affiliated organizations, or those of the publisher, the editors and the reviewers. Any product that may be evaluated in this article, or claim that may be made by its manufacturer, is not guaranteed or endorsed by the publisher.

## Supplementary material

Supplementary Data Sheet 1 Coding of t test/Mann-Whitney U rank test on numeric variables and Chi-squared Test/ Fisher Exact Test.

Supplementary Data Sheet 2 Coding of logistic regression.

Supplementary Data Sheet 3 Coding of factorial analysis.

Supplementary Data Sheet 4 Coding of scoring system.

Supplementary Data Sheet 5 Coding of exploratory data analysis.
