# Supplementary material for: Perinatal maternal characteristics predict a high risk of neonatal asphyxia: A multi-center retrospective cohort study in China
Source: Front Med (Lausanne). 2022 Aug 8;9:944272. doi: 10.3389/fmed.2022.944272 (PMC9393324; doi:10.3389/fmed.2022.944272)
Supplement: Supplementary Data Sheet 3 — Coding of factorial analysis. [file Data_Sheet_3.pdf]

FA

In [9]:

```python
import pandas as pd
from scipy import stats
from scipy.stats import fisher_exact
from scipy.stats import pearsonr
import pylab
import numpy as np

from sklearn.linear_model import LogisticRegression
from sklearn.model_selection import train_test_split
import statsmodels.api as sm
from sklearn import metrics
from sklearn.metrics import roc_auc_score,confusion_matrix,accuracy_score
from statsmodels.stats.outliers_influence import variance_inflation_factor
import matplotlib.pyplot as plt

import xgboost as xgb
import imblearn
from collections import Counter
from scipy.stats import pearsonr
pd.set_option('display.max_rows', 200)
from sklearn.preprocessing import normalize
#from factor_analyzer.factor_analyzer import FactorAnalyzer
from factor_analyzer import FactorAnalyzer
from factor_analyzer.factor_analyzer import calculate_bartlett_sphericity
```

In [2]:

```python
d0 = pd.read_csv('/Users/manzah/Code/yuyi/d0.xlsx',low_memory=False)
d1 = pd.read_csv('/Users/manzah/Code/yuyi/d1.xlsx',low_memory=False)
```

In [3]:

```python
# create target variable
d0['target'] = 0
d1['target'] = 1
```

In [4]:

```python
df = pd.concat([d0, d1])[['H3年龄','孕周','bmi','既往分娩次数','H13增重', 'H9胎数', 'F3_4_1破膜至分娩时间小时',
                         'F9_3新生儿1-体重','F2_1_0_4第二产程时间','F3_1妊娠期高血压1是', 'F3_4胎膜早破 1是',
                         'F3_5胎儿窘迫1是','F3_6脐带缠绕1是','F3_7脐带脱垂1是','F3_10产后出血1是','F4_1胎盘形态异常1是',
                         'F4_2前置胎盘1是', 'F4_4胎盘早剥1是', 'F4_6子宫破裂1是', 'F6_3合并症-心脏病1是',
                         'F6_4合并症-肝病1是', 'F6_8合并症-自身免疫病1是', 'F9_9新生儿1-复苏1是',
                         'F9_10新生儿1-产房气管插管1是', 'F6_2_1合并症-贫血', 'F9_6新生儿1-死胎/死亡','H8受孕方式 1自然2辅助生育
',
                         'F2_1分娩方式1VD2CS','F3_5_1胎儿窘迫1胎心型2羊水型','F9_1新生儿1-性别1男2女',
                         'F9_8新生儿1-结局 1出院2转儿科3死亡4放弃','F6_5_1合并症-糖尿病','F9_8_1新生儿1-转儿科','F7_1阴道分娩操
作',
                         'F2_1_1CS指征','阴道分娩','target']]
```

In [5]:

```python
#df['年龄'] = ['[45,)' if ele  >= 45 else '[40,45)' if ele >=40 else '[35,40)' if ele >= 35 else '35-' for ele in df['H
3年龄']]
df['年龄'] = ['[40,)' if ele  >= 40 else '(,40)' for ele in df['H3年龄']]
df['孕周3741'] = [ 1 if ele  > 41  else 1 if ele <37 else 0 for ele in df['孕周']]
df['阴道分娩'] = df['阴道分娩'].fillna('不适用')
df['既往分娩'] = [1 if ele  >= 1  else 0 for ele in df['既往分娩次数']]
#df['破膜至分娩时间小时'] = ['>48' if ele  > 48  else '24-48' if ele >24 else '12-24' if ele > 12 else '0-12' if ele>=0 e
lse '无' for ele in df['F3_4_1破膜至分娩时间小时']]
df['破膜至分娩时间小时'] = ['[48,)' if ele  > 48  else '(0,48]' if ele>=0 else '无' for ele in df['F3_4_1破膜至分娩时间小时'
]]
#df['第二产程时间'] = ['>4' if ele  > 4  else '2-4' if ele >2 else '0-2' if ele>=0 else '无' for ele in df['F2_1_0_4第二
产程时间']]
df['第二产程时间'] = ['(2,)' if ele  > 2  else '(0,2)' if ele >= 0 else '无' for ele in df['F2_1_0_4第二产程时间']]
df['新生儿1体重'] = ['>4000' if ele  > 4000  else '2500-4000' if ele >2500 else '<2500' for ele in df['F9_3新生儿1-体重']
]
#df['新生儿体重254'] = [1 if ele  > 4000  else 1 if ele < 2500 else 0 for ele in df['F9_3新生儿1-体重']]
```

In [7]:

```python
df = df[['既往分娩',
'F3_1妊娠期高血压1是',
'F3_5胎儿窘迫1是',
'F3_6脐带缠绕1是',
#'F4_2前置胎盘1是',
'孕周3741',
'年龄',
'破膜至分娩时间小时',
'新生儿1体重',

'F3_4胎膜早破 1是',
#'F3_7脐带脱垂1是',
'F4_1胎盘形态异常1是',

'阴道分娩',
#'F4_4胎盘早剥1是',
#'F4_6子宫破裂1是',
'F2_1分娩方式1VD2CS',
#'F3_5_1胎儿窘迫1胎心型2羊水型',
'第二产程时间',
'F7_1阴道分娩操作',
'F2_1_1CS指征',
'target']]
```

In [10]:

```python
X = df.drop(columns ='target' ) #(83294, 37)
y = df['target']
df_d = pd.get_dummies(X)  #(83294, 51)
```

In [12]:

```python
df_d = df_d[['既往分娩',
              'F3_1妊娠期高血压1是',
              'F3_5胎儿窘迫1是',
              'F3_6脐带缠绕1是', #'F4_2前置胎盘1是',
              '孕周3741', 'F3_4胎膜早破 1是',
             #'F3_7脐带脱垂1是',
             'F4_1胎盘形态异常1是', #'F4_4胎盘早剥1是',

             #'F4_6子宫破裂1是',
             'F2_1分娩方式1VD2CS', #'F3_5_1胎儿窘迫1胎心型2羊水型',
              '年龄_[40,)', '破膜至分娩时间小时_[48,)',
              '新生儿1体重_<2500', '新生儿1体重_>4000',
              '阴道分娩_胎吸/产钳',
              '第二产程时间_(2,)',
              'F7_1阴道分娩操作_引产/点滴加强/手转胎头',
              'F2_1_1CS指征_胎儿窘迫/难产']]
```

Bartlett's test

Bartlett's test of sphericity checks whether or not the observed variables intercorrelate at all using the observed correlation matrix against the identity matrix. If the test found statistically insignificant, you should not employ a factor analysis.

In [13]:

```python
chi_square_value,p_value=calculate_bartlett_sphericity(df_d)
chi_square_value, p_value
```

Out[13]:

```
(77775.55743639333, 0.0)
```

In this Bartlett 's test, the p-value is 0. The test was statistically significant, indicating that the observed correlation matrix is not an identity matrix.

Kaiser-Meyer-Olkin (KMO) Test

Kaiser-Meyer-Olkin (KMO) Test measures the suitability of data for factor analysis. It determines the adequacy for each observed variable and for the complete model. KMO estimates the proportion of variance among all the observed variable. Lower proportion is more suitable for factor analysis. KMO values range between 0 and 1.

In [ ]:

```python
from factor_analyzer.factor_analyzer import calculate_kmo
kmo_all,kmo_model=calculate_kmo(df_d)
```

In [17]:

```python
kmo_model
```

Out[17]:

```
0.5476299499025826
```

In [8]:

```python
#for var in X.columns:
#    print(var)
#    print(X[var].unique())
```

In [9]:

```python
pd.DataFrame({ 'Target Class':pd.value_counts(y, sort=True,normalize=True).index,
               'Count': pd.value_counts(y, sort=True).values,
               'Pct':pd.value_counts(y, sort=True,normalize=True).values})
```

Out[9]:

	Target Class	Count	Pct
0	0	82160	0.986386
1	1	1134	0.013614

In [9]:

```python
df_d.columns
```

Out[9]:

```
Index(['既往分娩', 'F3_1妊娠期高血压1是', 'F3_5胎儿窘迫1是', 'F3_6脐带缠绕1是', '孕周3741',
       'F3_4胎膜早破 1是', 'F4_1胎盘形态异常1是', 'F2_1分娩方式1VD2CS', '年龄_(,40)', '年龄_[40,)',
       '破膜至分娩时间小时_(0,48]', '破膜至分娩时间小时_[48,)', '破膜至分娩时间小时_无',
       '新生儿1体重_2500-4000', '新生儿1体重_<2500', '新生儿1体重_>4000', '阴道分娩_不适用',
       '阴道分娩_胎吸/产钳', '阴道分娩_自然', '第二产程时间_(0,2)', '第二产程时间_(2,)', '第二产程时间_无',
       'F7_1阴道分娩操作_other', 'F7_1阴道分娩操作_引产/点滴加强/手转胎头', 'F2_1_1CS指征_other',
       'F2_1_1CS指征_胎儿窘迫/难产'],
      dtype='object')
```

Factor Analysis

In [14]:

```python
data_scaled = normalize(df_d)
data_scaled = pd.DataFrame(data_scaled, columns=df_d.columns)
data_scaled.head()
```

Out[14]:

	既往分娩	F3_1妊娠期高血压1是	F3_5胎儿窘迫1是	F3_6脐带缠绕1是	孕周3741	F3_4胎膜早破1是	F4_1胎盘形态异常1是	F2_1分娩方式1VD2CS	年龄_[40,)	破膜至分娩时间小时_[48,)	新生儿1体重_<2500	新生儿1体重_>4000	阴道分娩_胎吸/产钳	第二产程时间_(2,)	F7_1阴道分娩操作_引产/点滴加强/手转胎头	F2_1_1CS指征_胎儿窘迫/难产
0	0.0	0.0	0.0	0.447214	0.0	0.0	0.0	0.894427	0.0	0.0	0.0	0.0	0.0	0.0	0.00000	0.0
1	0.0	0.0	0.0	0.577350	0.0	0.0	0.0	0.577350	0.0	0.0	0.0	0.0	0.0	0.0	0.57735	0.0
2	0.0	0.0	0.0	0.000000	0.0	0.0	0.0	1.000000	0.0	0.0	0.0	0.0	0.0	0.0	0.00000	0.0
3	0.0	0.0	0.0	0.000000	0.0	0.0	0.0	1.000000	0.0	0.0	0.0	0.0	0.0	0.0	0.00000	0.0
4	0.0	0.0	0.0	0.577350	0.0	0.0	0.0	0.577350	0.0	0.0	0.0	0.0	0.0	0.0	0.57735	0.0

- **Check Eigenvalues**

Eigenvalues represent variance explained each factor from the total variance. It is also known as characteristic roots.

The eigenvalue is a good criterion for determining the number of factors. Generally, an eigenvalue greater than 1 will be considered as selection criteria for the feature.

In [22]:

```python
fa = FactorAnalyzer(rotation='varimax',n_factors=3)
fa.fit(data_scaled)
# Check Eigenvalues
ev, v = fa.get_eigenvalues()
print(sum(ev > 1))
sorted(ev)
```

```
7
```

Out[22]:

```
[0.04303024612887324,
 0.7009308334004303,
 0.7103558138520765,
 0.7862966201486993,
 0.8267363569096196,
 0.8599677600979809,
 0.9211235831910968,
 0.948217824115751,
 0.9914463652293234,
 1.0177107836590595,
 1.0648892491960482,
 1.1422304915075414,
 1.1973832999781255,
 1.3628965892582408,
 1.4342131842555925,
 1.9925709990715337]
```

- **Factor loadings**

Factor loadings are similar to standardized regression coefficients, and variables with higher loadings on a particular factor can be interpreted as explaining a larger proportion of the variation in that factor.

The factor loading is a matrix which shows the relationship of each variable to the underlying factor. It shows the correlation coefficient for observed variable and factor. It shows the variance explained by the observed variables.

In [23]:

```python
loadings = pd.DataFrame(fa.loadings_,index = [data_scaled.columns])
loadings
```

Out[23]:

	0	1	2
既往分娩	-0.189698	0.981207	-0.089587
F3_1妊娠期高血压1是	-0.018525	-0.011024	0.165943
F3_5胎儿窘迫1是	0.226812	-0.110377	-0.012324
F3_6脐带缠绕1是	0.353154	0.004587	-0.060019
孕周3741	0.042294	-0.019413	0.497534
F3_4胎膜早破 1是	0.288541	-0.061715	0.070415
F4_1胎盘形态异常1是	0.230870	0.004364	-0.016571
F2_1分娩方式1VD2CS	-0.951800	-0.381362	-0.256539
年龄_[40,)	-0.011227	0.115773	0.001169
破膜至分娩时间小时_[48,)	0.104766	-0.030452	0.084048
新生儿1体重_<2500	-0.057749	0.008818	0.584015
新生儿1体重_>4000	0.055996	0.013793	-0.032512
阴道分娩_胎吸/产钳	0.188883	-0.029746	-0.012276
第二产程时间_(2,)	0.233581	0.182403	-0.024406
F7_1阴道分娩操作_引产/点滴加强/手转胎头	0.354623	-0.028976	-0.009532
F2_1_1CS指征_胎儿窘迫/难产	-0.012063	-0.149533	0.010992

In [18]:

```python
loadings.to_csv('loadings_varlist_7.csv')
```

In [19]:

```python
fa.get_factor_variance()
```

Out[19]:

```
(array([1.18121806, 1.15996737, 1.11062992, 1.10304873, 0.98902915,
        0.90082266, 0.69106387]),
 array([0.07382613, 0.07249796, 0.06941437, 0.06894055, 0.06181432,
        0.05630142, 0.04319149]),
 array([0.07382613, 0.14632409, 0.21573846, 0.284679  , 0.34649333,
        0.40279474, 0.44598623]))
```

Trivials

FAMD

https://pypi.org/project/prince/#factor-analysis-of-mixed-data-famd

In [16]:

```python
import prince
famd = prince.FAMD(n_components=2, n_iter=3,copy=True, check_input=True,engine='auto',random_state=42)
famd = famd.fit(X)
famd.row_coordinates(X)
```

Out[16]:

	0	1
0	0.604663	-0.371300
1	-1.167856	-1.108645
2	1.133012	-0.343531
3	0.773801	-0.461798
4	-1.223882	-1.037948
...	...	...
1129	0.796084	-0.133586
1130	-0.984419	-0.766231
1131	1.889861	0.512679
1132	1.972902	1.162157
1133	-0.797042	-1.071882

83294 rows × 2 columns

Resample

In [10]:

```python
from imblearn.over_sampling import SMOTE
from imblearn.under_sampling import RandomUnderSampler
from imblearn.over_sampling import RandomOverSampler
from imblearn.pipeline import Pipeline
from imblearn.over_sampling import SMOTENC
```

In [11]:

```python
# define pipeline
over = RandomOverSampler(sampling_strategy=0.1,random_state=1823)
under = RandomUnderSampler(sampling_strategy=0.5,random_state=1823)
steps = [('o', over), ('u', under)]
pipeline = Pipeline(steps=steps)
# transform the dataset
X_re, y_re = pipeline.fit_resample(X, y)

#
#under = RandomUnderSampler(sampling_strategy=0.1,random_state=1823)
#X_re1, y_re1 = under.fit_resample(X, y)
#smote_nc = SMOTENC(categorical_features=[0, 2, 3, 4, 5, 6, 7, 8, 9, 10, 11, 12, 13, 14],random_state=1823,sampling_st
rategy=0.5)
#X_re, y_re = smote_nc.fit_resample(X_re1, y_re1)
```

In [12]:

```python
pd.DataFrame({ 'Target Class':pd.value_counts(y_re, sort=True,normalize=True).index,
               'Count': pd.value_counts(y_re, sort=True).values,
               'Pct':pd.value_counts(y_re, sort=True,normalize=True).values})
```

Out[12]:

	Target Class	Count	Pct
0	0	16432	0.666667
1	1	8216	0.333333

Hierarchical Clustering

In [ ]:

```python
df_cont = X_re[['既往分娩', 'H13增重', 'F3_1妊娠期高血压1是', 'F3_5胎儿窘迫1是', 'F3_6脐带缠绕1是',
       'F4_2前置胎盘1是', 'F6_2_1合并症_贫血', 'H8受孕方式_1自然2辅助生育',
       '孕周3741', #'破膜至分娩时间小时', '第二产程时间', 'F6_5_1合并症_糖尿病','年龄', '新生儿1体重',
       '孕周']]
```

In [ ]:

```python
df_cont
```

In [ ]:

```python
data_scaled = normalize(df_cont)
data_scaled = pd.DataFrame(data_scaled, columns=df_cont.columns)
data_scaled.head()
```

In [18]:

```python
data_scaled.shape
```

Out[18]:

```
(24648, 10)
```

In [19]:

```python
import scipy.cluster.hierarchy as shc
plt.figure(figsize=(10, 7))
plt.title("Dendrograms")
dend = shc.dendrogram(shc.linkage(data_scaled, method='ward'))
```

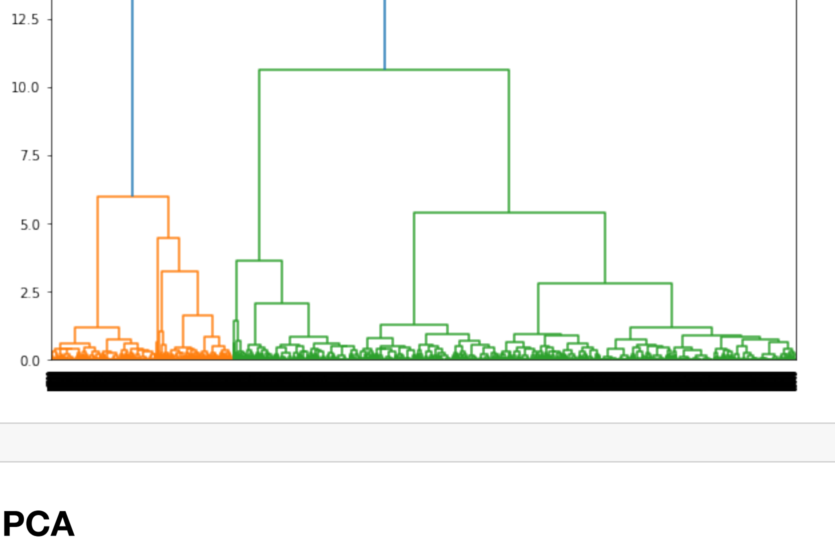

In [ ]:

PCA

In [20]:

```python
from sklearn.decomposition import PCA
```

In [21]:

```python
pca = PCA(n_components=2)
```

In [22]:

```python
principalComponents = pca.fit_transform(data_scaled)
```

In [23]:

```python
principalDf = pd.DataFrame(data = principalComponents, columns = ['principal component 1', 'principal component 2'])
```

In [25]:

```python
#finalDf = pd.concat([principalDf, df[['target']]], axis = 1)
```

In [ ]:
