# Supplementary material for: Perinatal maternal characteristics predict a high risk of neonatal asphyxia: A multi-center retrospective cohort study in China
Source: Front Med (Lausanne). 2022 Aug 8;9:944272. doi: 10.3389/fmed.2022.944272 (PMC9393324; doi:10.3389/fmed.2022.944272)
Supplement: Supplementary Data Sheet 4 — Coding of scoring system. [file Data_Sheet_4.pdf]

```python
In [1]: import pandas as pd
from scipy import stats
from scipy.stats import fisher_exact
from scipy.stats import pearsonr
import pylab
import numpy as np
import matplotlib.pyplot as plt

from sklearn.model_selection import train_test_split
from sklearn.metrics import roc_auc_score,confusion_matrix,accuracy_score,auc
pd.set_option('display.max_rows', 200)
```

```python
In [23]: df = pd.read_csv('/Users/manzah/Code/yuyi/分娩raw.xlsx',low_memory=False)
```

```python
In [24]: d = df[['F3_1妊娠期高血压1是','F3_5胎儿窘迫1是','F3_6脐带缠绕1是','F4_2前置胎盘1是','孕周',
        '破膜至分娩时间小时', '第二产程时间','新生儿1体重','target']]
```

Scoring Metrics

VAR	Score
破膜至分娩时间小时>=48	1
第二产程时间>=2	1
胎儿窘迫	4
妊娠期高血压	2
前置胎盘	2
脐带缠绕	1
孕周<37	2
新生儿1体重>=4000	1
新生儿1体重<2500	4

```python
In [ ]: score = d
#score['年龄'] = [1 if ele  == '[40,)' else 0 for ele in d['年龄']]
#score['既往分娩'] = [1 if ele  == 0 else 0 for ele in d['既往分娩']]
score['破膜至分娩时间小时'] = [1 if ele  == '[48,)' else 0 for ele in d['破膜至分娩时间小时']]
score['第二产程时间'] = [1 if ele  == '(2,)' else 0 for ele in d['第二产程时间']]
score['F3_5胎儿窘迫1是'] = [4 if ele  == 1 else 0 for ele in d['F3_5胎儿窘迫1是']]
score['F3_1妊娠期高血压1是'] = [2 if ele  == 1 else 0 for ele in d['F3_1妊娠期高血压1是']]
score['F4_2前置胎盘1是'] = [2 if ele  == 1 else 0 for ele in d['F4_2前置胎盘1是']]
score['F3_6脐带缠绕1是'] = [1 if ele  == 1 else 0 for ele in d['F3_6脐带缠绕1是']]
score['孕周'] = [2 if ele < 37 else 0 for ele in d['孕周']]
score['新生儿1体重'] = [1 if ele  == '>4000' else 4 if ele == '<2500' else 0 for ele in d['新生儿1体重']]


score['score']  = score[['破膜至分娩时间小时','第二产程时间','F3_5胎儿窘迫1是','F3_1妊娠期高血压1是',
                        'F4_2前置胎盘1是','F3_6脐带缠绕1是','孕周','新生儿1体重']].sum(axis=1)
```

```python
In [26]: score['score'].describe()
```

```
Out[26]: count    83294.000000
mean         1.144260
std          1.816774
min          0.000000
25%          0.000000
50%          0.000000
75%          1.000000
max         14.000000
Name: score, dtype: float64
```

Performance

Score system presentation (whole data)

```python
In [27]: t = pd.DataFrame(score[['target','score']].groupby('score').agg(['sum','count','mean']))
t.columns = t.columns.get_level_values(1)

ttlsum = np.cumsum(list(t['sum'])[::-1])[::-1]
ttlttl = np.cumsum(list(t['count'])[::-1])[::-1]
est_risk = np.round(ttlsum/ttlttl,3)
```

```python
In [28]: table = pd.DataFrame(list(zip(est_risk,list(t['sum']),list(t['count']),np.round(list(t['mean']),3),ttlsum,ttlttl)))
table.columns = ['est.risk','total number of death','total number of observation','death rate','sum of death','sum of total number of obs', ]
table
```

Out[28]:

	est.risk	total number of death	total number of observation	death rate	sum of death	sum of total number of obs
0	0.014	255	44314	0.006	1134	83294
1	0.023	203	19708	0.010	879	38980
2	0.035	124	6580	0.019	676	19272
3	0.043	50	1562	0.032	552	12692
4	0.045	145	4975	0.029	502	11130
5	0.058	107	2437	0.044	357	6155
6	0.067	108	2111	0.051	250	3718
7	0.088	36	514	0.070	142	1607
8	0.097	52	739	0.070	106	1093
9	0.153	13	161	0.081	54	354
10	0.212	23	123	0.187	41	193
11	0.257	7	34	0.206	18	70
12	0.306	7	28	0.250	11	36
13	0.500	3	6	0.500	4	8
14	0.500	1	2	0.500	1	2

Whole data

```python
In [29]: sensitivity = []
specificity = []
accuracy = []
cutoff = []
target = score['target']
for c in range(0,19):
    prediction = [1 if ele  >= c  else 0 for ele in score['score']]
    #roc x=1-specificity, y=sensitivity
    tn, fp, fn, tp = confusion_matrix(target, prediction).ravel()
    specificity.append(tn / (tn+fp))
    sensitivity.append(tp / (tp+fn))
    accuracy.append((tp + tn) / (tp + tn + fp + fn))
    cutoff.append(c)
```

```python
In [30]: fpr = [1-x for x in specificity]
results = pd.DataFrame(list(zip(cutoff,sensitivity,specificity,accuracy)))
results.columns = ['cut-off','sensitivity', 'specificity','accuracy']
results
```

Out[30]:

	cut-off	sensitivity	specificity	accuracy
0	0	1.000000	0.000000	0.013614
1	1	0.775132	0.536259	0.539511
2	2	0.596120	0.773661	0.771244
3	3	0.486772	0.852240	0.847264
4	4	0.442681	0.870643	0.864816
5	5	0.314815	0.929430	0.921063
6	6	0.220459	0.957790	0.947751
7	7	0.125220	0.982169	0.970502
8	8	0.093474	0.987987	0.975809
9	9	0.047619	0.996349	0.983432
10	10	0.036155	0.998150	0.985053
11	11	0.015873	0.999367	0.985977
12	12	0.009700	0.999696	0.986217
13	13	0.003527	0.999951	0.986386
14	14	0.000882	0.999988	0.986386
15	15	0.000000	1.000000	0.986386
16	16	0.000000	1.000000	0.986386
17	17	0.000000	1.000000	0.986386
18	18	0.000000	1.000000	0.986386

- ROC curve

```python
In [31]: print('c stat = ' + str(auc(fpr,sensitivity)))
```

```
c stat = 0.7252413022982642
```

```python
In [32]: plt.figure(dpi=300)
#plt.figure(figsize=(8,6))

plt.plot(fpr,sensitivity)
plt.xlabel("False Positive Rate")
plt.ylabel("True Positive Rate")
plt.xlim([0.0, 1.0])
plt.ylim([0.0, 1.05])
plt.plot([0, 1], [0, 1], "k--", lw=2)
plt.show()
```

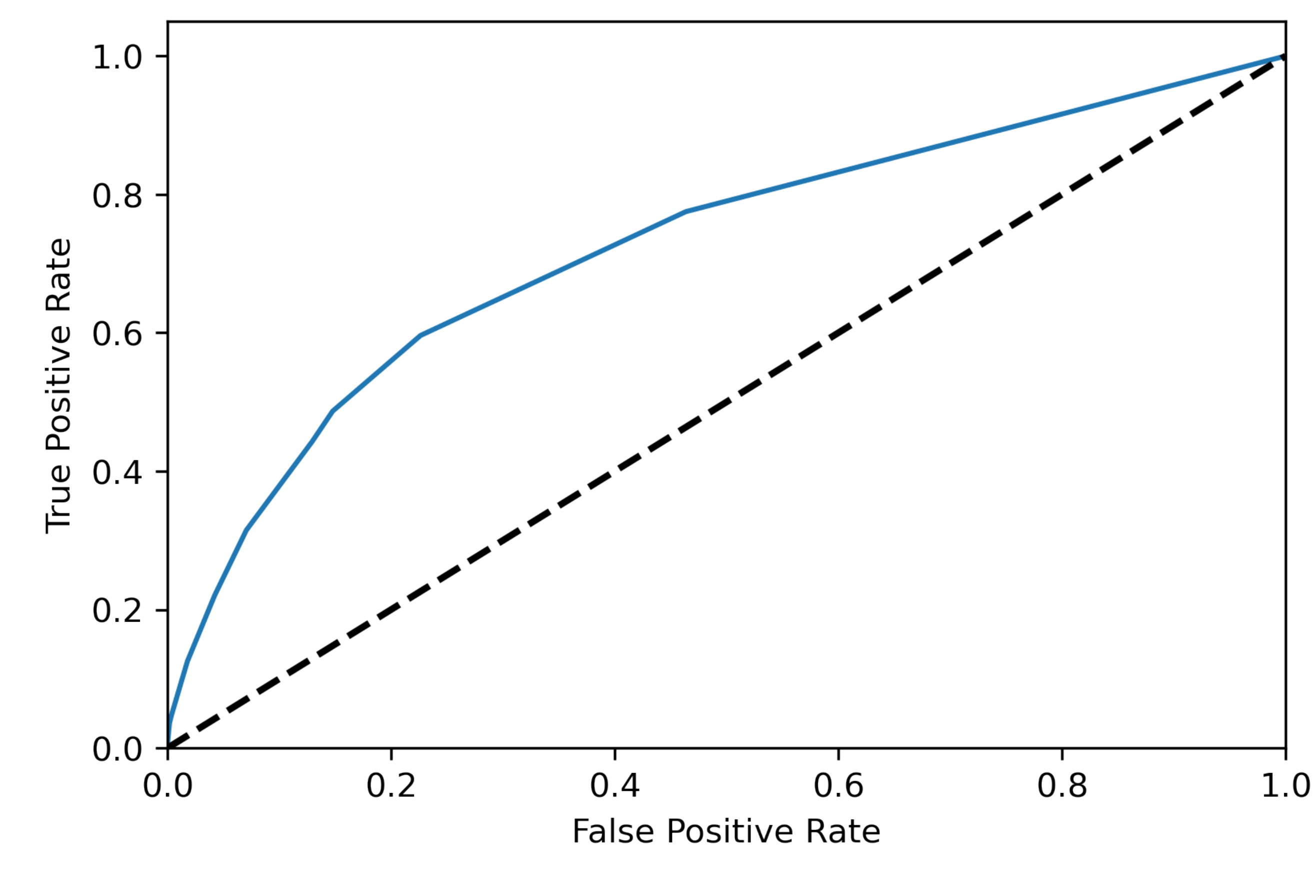

Training and est

```python
In [33]: X = score.drop(columns ='target' )
y = score['target']
X_train, X_test, y_train, y_test = train_test_split(X, y, test_size=0.2, random_state=107)
```

- Training

```python
In [34]: X_train['score'].describe()
```

```
Out[34]: count    66635.000000
mean         1.140692
std          1.816044
min          0.000000
25%          0.000000
50%          0.000000
75%          1.000000
max         14.000000
Name: score, dtype: float64
```

```python
In [35]: sensitivity_tr = []
specificity_tr = []
cutoff_tr = []
accuracy_tr = []
for c in range(0,19):
    prediction = [1 if ele  >= c  else 0 for ele in X_train['score']]
    #roc x=1-specificity, y=sensitivity
    tn, fp, fn, tp = confusion_matrix(y_train, prediction).ravel()
    specificity_tr.append(tn / (tn+fp))
    sensitivity_tr.append(tp / (tp+fn))
    accuracy_tr.append((tp + tn) / (tp + tn + fp + fn))
    cutoff_tr.append(c)

fpr_tr = [1-x for x in specificity_tr]
results_tr = pd.DataFrame(list(zip(cutoff,sensitivity_tr,specificity_tr,accuracy_tr)))
results_tr.columns = ['cutoff_tr','sensitivity_tr', 'specificity_tr','accuracy_tr']
results_tr
```

Out[35]:

	cutoff_tr	sensitivity_tr	specificity_tr	accuracy_tr
0	0	1.000000	0.000000	0.013236
1	1	0.776644	0.536979	0.540152
2	2	0.586168	0.774946	0.772447
3	3	0.479592	0.853026	0.848083
4	4	0.433107	0.870941	0.865146
5	5	0.314059	0.929053	0.920912
6	6	0.213152	0.957781	0.947925
7	7	0.120181	0.981948	0.970541
8	8	0.086168	0.987712	0.975778
9	9	0.041950	0.996304	0.983672
10	10	0.031746	0.998084	0.985293
11	11	0.015873	0.999346	0.986329
12	12	0.009070	0.999681	0.986569
13	13	0.003401	0.999954	0.986764
14	14	0.000000	0.999985	0.986749
15	15	0.000000	1.000000	0.986764
16	16	0.000000	1.000000	0.986764
17	17	0.000000	1.000000	0.986764
18	18	0.000000	1.000000	0.986764

- Test

```python
In [36]: X_test['score'].describe()
```

```
Out[36]: count    16659.000000
mean         1.158533
std          1.819676
min          0.000000
25%          0.000000
50%          0.000000
75%          1.000000
max         14.000000
Name: score, dtype: float64
```

```python
In [37]: sensitivity_test = []
specificity_test = []
cutoff_test = []
accuracy_test = []
for c in range(0,18): #0,1,...,21,22
    prediction = [1 if ele  >= c  else 0 for ele in X_test['score']]
    #roc x=1-specificity, y=sensitivity
    tn, fp, fn, tp = confusion_matrix(y_test, prediction).ravel()
    specificity_test.append(tn / (tn+fp))
    sensitivity_test.append(tp / (tp+fn))
    accuracy_test.append((tp + tn) / (tp + tn + fp + fn))
    cutoff_test.append(c)

fpr_test = [1-x for x in specificity_test]
results_test = pd.DataFrame(list(zip(cutoff,sensitivity_test,specificity_test,accuracy_test)))
results_test.columns = ['cutoff_test','sensitivity_test', 'specificity_test','accuracy_test']
results_test
```

Out[37]:

	cutoff_test	sensitivity_test	specificity_test	accuracy_test
0	0	1.000000	0.000000	0.015127
1	1	0.769841	0.533370	0.536947
2	2	0.630952	0.768513	0.766433
3	3	0.511905	0.849089	0.843988
4	4	0.476190	0.869446	0.863497
5	5	0.317460	0.930944	0.921664
6	6	0.246032	0.957823	0.947056
7	7	0.142857	0.983056	0.970346
8	8	0.119048	0.989090	0.975929
9	9	0.067460	0.996526	0.982472
10	10	0.051587	0.998415	0.984093
11	11	0.015873	0.999451	0.984573
12	12	0.011905	0.999756	0.984813
13	13	0.003968	0.999939	0.984873
14	14	0.003968	1.000000	0.984933
15	15	0.000000	1.000000	0.984873
16	16	0.000000	1.000000	0.984873
17	17	0.000000	1.000000	0.984873

- **ROC curve**

```python
In [38]: plt.figure(dpi=300)

plt.plot(fpr_tr,sensitivity_tr,label='Training ROC Curve')
plt.plot(fpr_test,sensitivity_test,label="Test ROC Curve")


plt.legend(loc=4)
plt.xlabel("False Positive Rate")
plt.ylabel("True Positive Rate")
plt.xlim([0.0, 1.0])
plt.ylim([0.0, 1.05])
plt.plot([0, 1], [0, 1], "k--", lw=2)
plt.show()
```

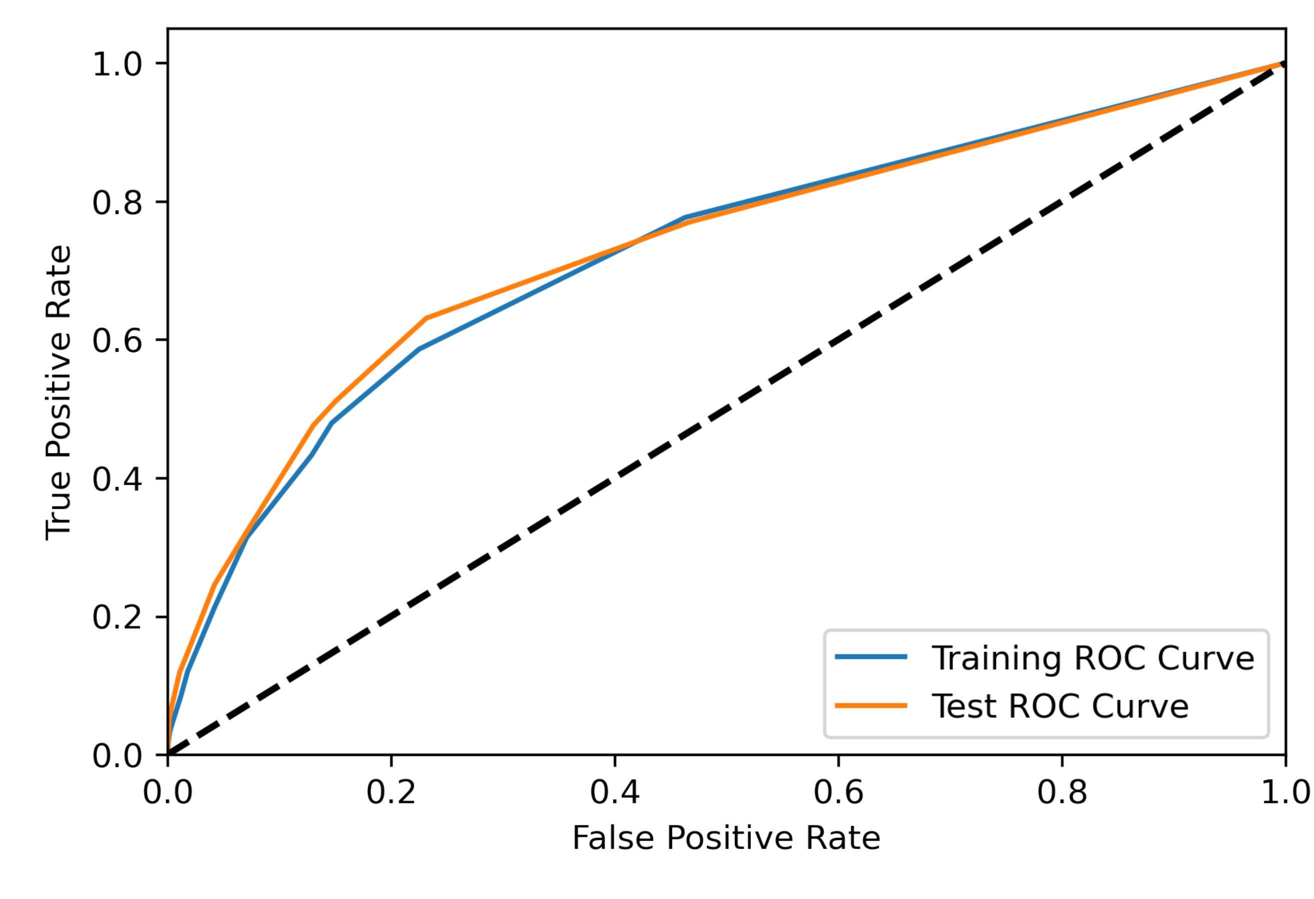


```python
In [39]: print(auc(fpr,sensitivity))
print(auc(fpr_tr,sensitivity_tr))
print(auc(fpr_test,sensitivity_test))
```

```
0.7252413022982642
0.7235020927801921
0.7313046792842002
```

```python
In [ ]:
```

```python
In [ ]:
```
