# Supplementary material for: Perinatal maternal characteristics predict a high risk of neonatal asphyxia: A multi-center retrospective cohort study in China
Source: Front Med (Lausanne). 2022 Aug 8;9:944272. doi: 10.3389/fmed.2022.944272 (PMC9393324; doi:10.3389/fmed.2022.944272)
Supplement: Supplementary Data Sheet 1 — Coding of t test/Mann-Whitney U rank test on numeric variables and Chi-squared Test/ Fisher Exact Test. [file Data_Sheet_1.pdf]

In [2]:
```python
import pandas as pd
from scipy import stats
from scipy.stats import fisher_exact
from scipy.stats import pearsonr
import pylab
import numpy as np

from sklearn.linear_model import LogisticRegression
from sklearn.model_selection import train_test_split
import statsmodels.api as sm
from sklearn import metrics
from sklearn.metrics import roc_auc_score,confusion_matrix,accuracy_score
from statsmodels.stats.outliers_influence import variance_inflation_factor
import matplotlib.pyplot as plt
from statsmodels.sandbox.stats.multicomp import multipletests

pd.set_option('display.max_rows', 200)
```

In [3]:
```python
d0 = pd.read_csv('/Users/manzah/Code/yuyi/d0.xlsx',low_memory=False)
d1 = pd.read_csv('/Users/manzah/Code/yuyi/d1.xlsx',low_memory=False)
```

窒息

In [7]:
```python
import statistics

num = ['H3年龄','孕周','bmi','既往分娩次数','H13增重', 'H9胎数', 'F3_4_1破膜至分娩时间小时','F9_3新生儿1-体重','F2_1_0_4第二产程时间']

table = pd.DataFrame()
mean  = []
median = []
std = []
for var in num:

    table['var'] = var

    mean.append(round(statistics.mean(d1[var].dropna()),3))
    median.append(round(statistics.median(d1[var].dropna()),3))
    std.append(round(statistics.stdev(d1[var].dropna()),3))

    #print(round(statistics.mean(amn[var].dropna()),3))
    #print(round(statistics.median(amn[var].dropna()),3))
    #print(round(statistics.stdev(amn[var].dropna()),3))
```

In [8]:
```python
df = pd.DataFrame({'variable':num, 'mean':mean, 'median': median, 'std':std})
df
```

Out[8]:

	variable	mean	median	std
0	H3年龄	30.050	29.000	4.938
1	孕周	38.299	38.714	2.061
2	bmi	27.140	27.010	4.041
3	既往分娩次数	0.381	0.000	0.521
4	H13增重	15.579	15.000	5.446
5	H9胎数	1.036	1.000	0.187
6	F3_4_1破膜至分娩时间小时	20.203	13.000	24.006
7	F9_3新生儿1-体重	3078.917	3120.000	662.788
8	F2_1_0_4第二产程时间	1.081	0.767	0.915

非窒息

In [9]:
```python
import statistics

num = ['H3年龄','孕周','bmi','既往分娩次数','H13增重', 'H9胎数', 'F3_4_1破膜至分娩时间小时','F9_3新生儿1-体重','F2_1_0_4第二产程时间']

table = pd.DataFrame()
mean  = []
median = []
std = []
for var in num:

    table['var'] = var

    mean.append(round(statistics.mean(d0[var].dropna()),3))
    median.append(round(statistics.median(d0[var].dropna()),3))
    std.append(round(statistics.stdev(d0[var].dropna()),3))


df = pd.DataFrame({'variable':num, 'mean':mean, 'median': median, 'std':std})
df
```

Out[9]:

	variable	mean	median	std
0	H3年龄	30.728	30.000	4.514
1	孕周	39.060	39.143	1.410
2	bmi	26.803	26.446	8.093
3	既往分娩次数	0.420	0.000	0.520
4	H13增重	15.567	15.000	5.250
5	H9胎数	1.028	1.000	0.165
6	F3_4_1破膜至分娩时间小时	18.006	12.000	33.185
7	F9_3新生儿1-体重	3300.266	3300.000	463.898
8	F2_1_0_4第二产程时间	0.761	0.550	12.913

t test/Mann-Whitney U rank test on numeric variables

Mann-Whitney U rank test = ['孕周', '既往分娩次数', 'H9胎数', 'F3_4_1破膜至分娩时间小时', 'F2_1_0_4第二产程时间']

In [86]:
```python
p = stats.ttest_ind(d1['H3年龄'], d0['H3年龄'])[1]
p_adjusted = multipletests(p, alpha=0.05, method='bonferroni')
print('T test bonferroni corrected p value is ' + str(p_adjusted[1]))
print('bonferroni corrected alpha is ' + str(p_adjusted[3]))
```
```
T test bonferroni corrected p value is [5.27131775e-07]
bonferroni corrected alpha is 0.05
```

In [87]:
```python
#stats.ttest_ind(d1['孕周'], d0['孕周'])
U1, p = stats.mannwhitneyu(d0['孕周'], d1['孕周'])
p_adjusted = multipletests(p, alpha=0.05, method='bonferroni')
print('Mann-Whitney U rank test bonferroni corrected p value is ' + str(p_adjusted[1]))
print('bonferroni corrected alpha is ' + str(p_adjusted[3]))
```
```
Mann-Whitney U rank test bonferroni corrected p value is [3.20724119e-26]
bonferroni corrected alpha is 0.05
```

In [88]:
```python
p = stats.ttest_ind(d1['bmi'], d0['bmi'])[1]
p_adjusted = multipletests(p, alpha=0.05, method='bonferroni')
print('T test bonferroni corrected p value is ' + str(p_adjusted[1]))
print('bonferroni corrected alpha is ' + str(p_adjusted[3]))
```
```
T test bonferroni corrected p value is [0.16169106]
bonferroni corrected alpha is 0.05
```

In [89]:
```python
#p = stats.ttest_ind(d1['既往分娩次数'], d0['既往分娩次数'])
U1, p = stats.mannwhitneyu(d0['既往分娩次数'], d1['既往分娩次数'])
p_adjusted = multipletests(p, alpha=0.05, method='bonferroni')
print('Mann-Whitney U rank test bonferroni corrected p value is ' + str(p_adjusted[1]))
print('bonferroni corrected alpha is ' + str(p_adjusted[3]))
```
```
Mann-Whitney U rank test bonferroni corrected p value is [0.00286481]
bonferroni corrected alpha is 0.05
```

In [90]:
```python
p = stats.ttest_ind(d1['H13增重'], d0['H13增重'])[1]
p_adjusted = multipletests(p, alpha=0.05, method='bonferroni')
print('T test bonferroni corrected p value is ' + str(p_adjusted[1]))
print('bonferroni corrected alpha is ' + str(p_adjusted[3]))
```
```
T test bonferroni corrected p value is [0.93655176]
bonferroni corrected alpha is 0.05
```

In [91]:
```python
p = stats.ttest_ind(d1['H9胎数'], d0['H9胎数'])[1]
p_adjusted = multipletests(p, alpha=0.05, method='bonferroni')
print('T test bonferroni corrected p value is ' + str(p_adjusted[1]))
print('bonferroni corrected alpha is ' + str(p_adjusted[3]))
```
```
T test bonferroni corrected p value is [0.08798488]
bonferroni corrected alpha is 0.05
```

In [92]:
```python
U1, p = stats.mannwhitneyu(d0['H9胎数'], d1['H9胎数'])
p_adjusted = multipletests(p, alpha=0.05, method='bonferroni')
print('Mann-Whitney U rank bonferroni corrected test p value is ' + str(p_adjusted[1]))
print('bonferroni corrected alpha is ' + str(p_adjusted[3]))
```
```
Mann-Whitney U rank bonferroni corrected test p value is [0.04207207]
bonferroni corrected alpha is 0.05
```

In [93]:
```python
#stats.ttest_ind(d1['F3_4_1破膜至分娩时间小时'].dropna(), d0['F3_4_1破膜至分娩时间小时'].dropna())
U1, p = stats.mannwhitneyu(d0['F3_4_1破膜至分娩时间小时'].dropna(), d1['F3_4_1破膜至分娩时间小时'].dropna())
p_adjusted = multipletests(p, alpha=0.05, method='bonferroni')
print('Mann-Whitney U rank bonferroni corrected test p value is ' + str(p_adjusted[1]))
print('bonferroni corrected alpha is ' + str(p_adjusted[3]))
```
```
Mann-Whitney U rank bonferroni corrected test p value is [0.14744453]
bonferroni corrected alpha is 0.05
```

In [94]:
```python
p = stats.ttest_ind(d1['F9_3新生儿1-体重'], d0['F9_3新生儿1-体重'])[1]
p_adjusted = multipletests(p, alpha=0.05, method='bonferroni')
print('T test bonferroni corrected p value is ' + str(p_adjusted[1]))
print('bonferroni corrected alpha is ' + str(p_adjusted[3]))
```
```
T test bonferroni corrected p value is [1.80039043e-56]
bonferroni corrected alpha is 0.05
```

In [95]:
```python
#stats.ttest_ind(d1['F2_1_0_4第二产程时间'].dropna(), d0['F2_1_0_4第二产程时间'].dropna())
U1, p = stats.mannwhitneyu(d0['F2_1_0_4第二产程时间'].dropna(), d1['F2_1_0_4第二产程时间'].dropna())
p_adjusted = multipletests(p, alpha=0.05, method='bonferroni')
print('T test bonferroni corrected p value is ' + str(p_adjusted[1]))
print('bonferroni corrected alpha is ' + str(p_adjusted[3]))
```
```
T test bonferroni corrected p value is [1.74170759e-16]
bonferroni corrected alpha is 0.05
```

Chi-squred Test/ Fisher Exact Test¶

In [8]:
```python
binary = [ 'F3_1妊娠期高血压1是', 'F3_4胎膜早破 1是','F3_5胎儿窘迫1是','F3_6脐带缠绕1是','F3_7脐带脱垂1是',
          'F3_10产后出血1是','F4_1胎盘形态异常1是','F4_2前置胎盘1是', 'F4_4胎盘早剥1是', 'F4_6子宫破裂1是',
          'F6_3合并症-心脏病1是','F6_4合并症-肝病1是', 'F6_8合并症-自身免疫病1是', 'F9_9新生儿1-复苏1是',
          'F9_10新生儿1-产房气管插管1是', 'F6_2_1合并症-贫血', 'F9_6新生儿1-死胎/死亡',]
for var in binary:
    obs = np.array([[sum(d0[var]==1), sum(d1[var]==1)],
                    [sum(d0[var]==0), sum(d1[var]==0)]])
    chi2, p, dof, ex = stats.chi2_contingency(obs)
    p_adjusted = multipletests(p, alpha=0.05, method='bonferroni')
    print(var + ' bonferroni corrected p-value is ' + str(p_adjusted[1]) + ' and bonferroni corrected alpha is ' + str
(p_adjusted[3]) )
```
```
F3_1妊娠期高血压1是 bonferroni corrected p-value is [1.63215179e-28] and bonferroni corrected alpha is 0.05
F3_4胎膜早破 1是 bonferroni corrected p-value is [0.00024711] and bonferroni corrected alpha is 0.05
F3_5胎儿窘迫1是 bonferroni corrected p-value is [4.24901314e-122] and bonferroni corrected alpha is 0.05
F3_6脐带缠绕1是 bonferroni corrected p-value is [1.31094461e-14] and bonferroni corrected alpha is 0.05
F3_7脐带脱垂1是 bonferroni corrected p-value is [2.30250348e-12] and bonferroni corrected alpha is 0.05
F3_10产后出血1是 bonferroni corrected p-value is [2.81523965e-06] and bonferroni corrected alpha is 0.05
F4_1胎盘形态异常1是 bonferroni corrected p-value is [8.79425848e-12] and bonferroni corrected alpha is 0.05
F4_2前置胎盘1是 bonferroni corrected p-value is [5.55229301e-19] and bonferroni corrected alpha is 0.05
F4_4胎盘早剥1是 bonferroni corrected p-value is [7.87111581e-28] and bonferroni corrected alpha is 0.05
F4_6子宫破裂1是 bonferroni corrected p-value is [0.03865059] and bonferroni corrected alpha is 0.05
F6_3合并症-心脏病1是 bonferroni corrected p-value is [0.00010969] and bonferroni corrected alpha is 0.05
F6_4合并症-肝病1是 bonferroni corrected p-value is [0.00046258] and bonferroni corrected alpha is 0.05
F6_8合并症-自身免疫病1是 bonferroni corrected p-value is [0.01269071] and bonferroni corrected alpha is 0.05
F9_9新生儿1-复苏1是 bonferroni corrected p-value is [0.] and bonferroni corrected alpha is 0.05
F9_10新生儿1-产房气管插管1是 bonferroni corrected p-value is [0.] and bonferroni corrected alpha is 0.05
F6_2_1合并症-贫血 bonferroni corrected p-value is [0.85493307] and bonferroni corrected alpha is 0.05
F9_6新生儿1-死胎/死亡 bonferroni corrected p-value is [0.68433708] and bonferroni corrected alpha is 0.05
```

In [9]:
```python
obs = np.array([[sum(d0['H8受孕方式 1自然2辅助生育']==1), sum(d1['H8受孕方式 1自然2辅助生育']==1)],
                [sum(d0['H8受孕方式 1自然2辅助生育']==2), sum(d1['H8受孕方式 1自然2辅助生育']==2)]])
chi2, p, dof, ex = stats.chi2_contingency(obs)
p_adjusted = multipletests(p, alpha=0.05, method='bonferroni')
print('bonferroni corrected p-value is ' + str(p_adjusted[1]) + ' and bonferroni corrected alpha is ' + str(p_adjusted
[3]) )
```
```
bonferroni corrected p-value is [0.459501] and bonferroni corrected alpha is 0.05
```

In [10]:
```python
obs = np.array([[sum(d0['F2_1分娩方式1VD2CS']==1), sum(d1['F2_1分娩方式1VD2CS']==1)],
                [sum(d0['F2_1分娩方式1VD2CS']==2), sum(d1['F2_1分娩方式1VD2CS']==2)]])
chi2, p, dof, ex = stats.chi2_contingency(obs)
p_adjusted = multipletests(p, alpha=0.05, method='bonferroni')
print('bonferroni corrected p-value is ' + str(p_adjusted[1]) + ' and bonferroni corrected alpha is ' + str(p_adjusted
[3]) )
```
```
bonferroni corrected p-value is [0.00022881] and bonferroni corrected alpha is 0.05
```

In [11]:
```python
obs = np.array([[sum(d0['F3_5_1胎儿窘迫1胎心型2羊水型']==1), sum(d1['F3_5_1胎儿窘迫1胎心型2羊水型']==1)],
                [sum(d0['F3_5_1胎儿窘迫1胎心型2羊水型']==2), sum(d1['F3_5_1胎儿窘迫1胎心型2羊水型']==2)],
               [sum(d0['F3_5_1胎儿窘迫1胎心型2羊水型']==0), sum(d1['F3_5_1胎儿窘迫1胎心型2羊水型']==0)]])
chi2, p, dof, ex = stats.chi2_contingency(obs)
p_adjusted = multipletests(p, alpha=0.05, method='bonferroni')
print('bonferroni corrected p-value is ' + str(p_adjusted[1]) + ' and bonferroni corrected alpha is ' + str(p_adjusted
[3]) )
```
```
bonferroni corrected p-value is [2.66470744e-123] and bonferroni corrected alpha is 0.05
```

In [12]:
```python
obs = np.array([[sum(d0['F9_1新生儿1-性别1男2女']==1), sum(d1['F9_1新生儿1-性别1男2女']==1)],
                [sum(d0['F9_1新生儿1-性别1男2女']==2), sum(d1['F9_1新生儿1-性别1男2女']==2)]])
chi2, p, dof, ex = stats.chi2_contingency(obs)
p_adjusted = multipletests(p, alpha=0.05, method='bonferroni')
print('bonferroni corrected p-value is ' + str(p_adjusted[1]) + ' and bonferroni corrected alpha is ' + str(p_adjusted
[3]) )
```
```
bonferroni corrected p-value is [0.04152642] and bonferroni corrected alpha is 0.05
```

In [13]:
```python
obs = np.array([[sum(d0['F9_8新生儿1-结局 1出院2转儿科3死亡4放弃']==1), sum(d1['F9_8新生儿1-结局 1出院2转儿科3死亡4放弃']==1)],
                [sum(d0['F9_8新生儿1-结局 1出院2转儿科3死亡4放弃']==2), sum(d1['F9_8新生儿1-结局 1出院2转儿科3死亡4放弃']==2)],
                [sum(d0['F9_8新生儿1-结局 1出院2转儿科3死亡4放弃']==3), sum(d1['F9_8新生儿1-结局 1出院2转儿科3死亡4放弃']==3)],
                [sum(d0['F9_8新生儿1-结局 1出院2转儿科3死亡4放弃']==4), sum(d1['F9_8新生儿1-结局 1出院2转儿科3死亡4放弃']==4)]]
)
chi2, p, dof, ex = stats.chi2_contingency(obs)
p_adjusted = multipletests(p, alpha=0.05, method='bonferroni')
print('bonferroni corrected p-value is ' + str(p_adjusted[1]) + ' and bonferroni corrected alpha is ' + str(p_adjusted
[3]) )
```
```
bonferroni corrected p-value is [0.] and bonferroni corrected alpha is 0.05
```

In [14]:
```python
obs = np.array([[sum(d0['F6_5_1合并症-糖尿病']=='0'), sum(d1['F6_5_1合并症-糖尿病']=='0')],
                [sum(d0['F6_5_1合并症-糖尿病']=='GDM'), sum(d1['F6_5_1合并症-糖尿病']=='GDM')],
                [sum(d0['F6_5_1合并症-糖尿病']=='I型/II型'), sum(d1['F6_5_1合并症-糖尿病']=='I型/II型')]])
chi2, p, dof, ex = stats.chi2_contingency(obs)
p_adjusted = multipletests(p, alpha=0.05, method='bonferroni')
print('bonferroni corrected p-value is ' + str(p_adjusted[1]) + ' and bonferroni corrected alpha is ' + str(p_adjusted
[3]) )
```
```
bonferroni corrected p-value is [9.41071905e-36] and bonferroni corrected alpha is 0.05
```

In [15]:
```python
obs = np.array([[sum(d0['F9_8_1新生儿1-转儿科']=='other'), sum(d1['F9_8_1新生儿1-转儿科']=='other')],
                [sum(d0['F9_8_1新生儿1-转儿科']=='早产/感染/窒息/RDS/吸入性肺炎/湿肺'),
                 sum(d1['F9_8_1新生儿1-转儿科']=='早产/感染/窒息/RDS/吸入性肺炎/湿肺')]])
chi2, p, dof, ex = stats.chi2_contingency(obs)
p_adjusted = multipletests(p, alpha=0.05, method='bonferroni')
print('bonferroni corrected p-value is ' + str(p_adjusted[1]) + ' and bonferroni corrected alpha is ' + str(p_adjusted
[3]) )
```
```
bonferroni corrected p-value is [0.] and bonferroni corrected alpha is 0.05
```

In [16]:
```python
obs = np.array([[sum(d0['F7_1阴道分娩操作']=='other'), sum(d1['F7_1阴道分娩操作']=='other')],
                [sum(d0['F7_1阴道分娩操作']=='引产/点滴加强/手转胎头'), sum(d1['F7_1阴道分娩操作']=='引产/点滴加强/手转胎头')]])
chi2, p, dof, ex = stats.chi2_contingency(obs)
p_adjusted = multipletests(p, alpha=0.05, method='bonferroni')
print('bonferroni corrected p-value is ' + str(p_adjusted[1]) + ' and bonferroni corrected alpha is ' + str(p_adjusted
[3]) )
```
```
bonferroni corrected p-value is [0.25836038] and bonferroni corrected alpha is 0.05
```

In [17]:
```python
obs = np.array([[sum(d0['F2_1_1CS指征']=='other'), sum(d1['F2_1_1CS指征']=='other')],
                [sum(d0['F2_1_1CS指征']=='胎儿窘迫/难产'), sum(d1['F2_1_1CS指征']=='胎儿窘迫/难产')]])
chi2, p, dof, ex = stats.chi2_contingency(obs)
p_adjusted = multipletests(p, alpha=0.05, method='bonferroni')
print('bonferroni corrected p-value is ' + str(p_adjusted[1]) + ' and bonferroni corrected alpha is ' + str(p_adjusted
[3]) )
```
```
bonferroni corrected p-value is [4.21997256e-32] and bonferroni corrected alpha is 0.05
```

In [18]:
```python
obs = np.array([[sum(d0['阴道分娩']=='自然'), sum(d1['阴道分娩']=='自然')],
                [sum(d0['阴道分娩']=='胎吸/产钳'), sum(d1['阴道分娩']=='胎吸/产钳')],
                [d0['阴道分娩'].isnull().sum(),d1['阴道分娩'].isnull().sum()]])
chi2, p, dof, ex = stats.chi2_contingency(obs)
p_adjusted = multipletests(p, alpha=0.05, method='bonferroni')
print('bonferroni corrected p-value is ' + str(p_adjusted[1]) + ' and bonferroni corrected alpha is ' + str(p_adjusted
[3]) )
```
```
bonferroni corrected p-value is [1.62549979e-65] and bonferroni corrected alpha is 0.05
```

normality check

In [5]:
```python
cont = ['H3年龄','孕周','bmi',
        '既往分娩次数','H13增重',
        'H9胎数','F3_4_1破膜至分娩时间小时',
        'F9_3新生儿1-体重','F2_1_0_4第二产程时间']
```

In [14]:
```python
prob = ['孕周',
        '既往分娩次数',
        'H9胎数',
        'F3_4_1破膜至分娩时间小时',
        'F2_1_0_4第二产程时间']
for var in prob:
    stats.probplot(d1[var], dist="norm", plot=pylab)
    pylab.show()
```

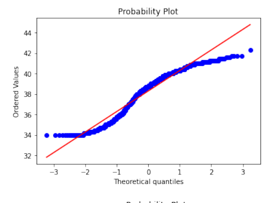
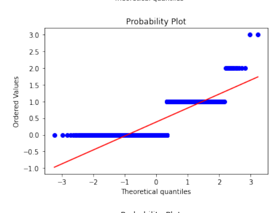
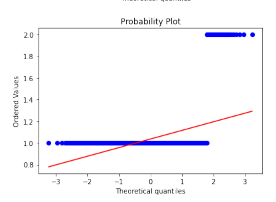
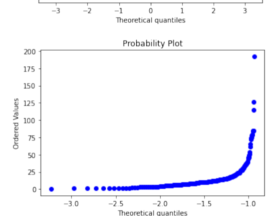
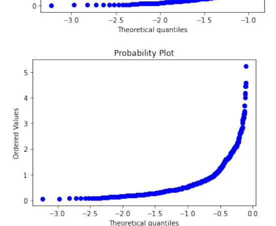

In [10]:
```python
for var in cont:
    stats.probplot(d1[var], dist="norm", plot=pylab)
    pylab.show()
```

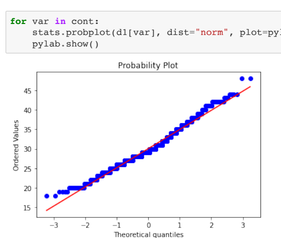
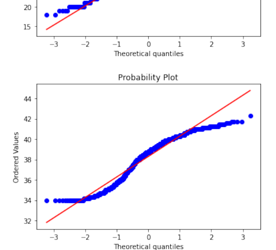
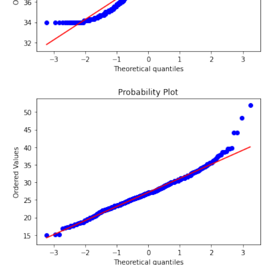
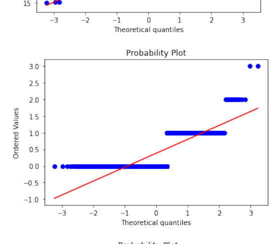
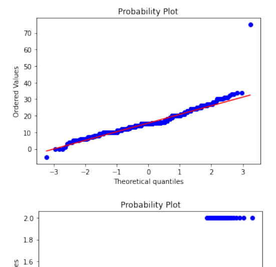
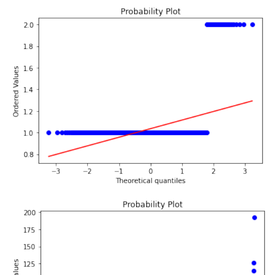
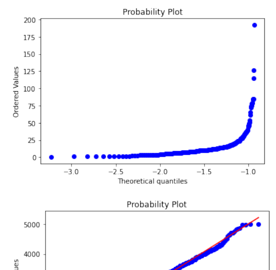
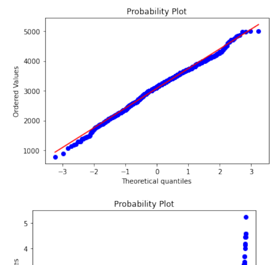
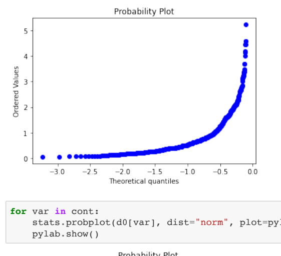

In [6]:
```python
for var in cont:
    stats.probplot(d0[var], dist="norm", plot=pylab)
    pylab.show()
```

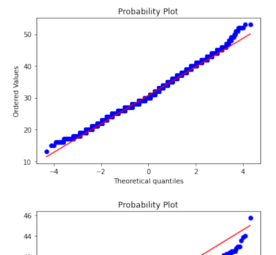
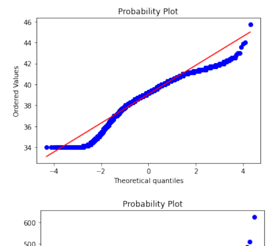
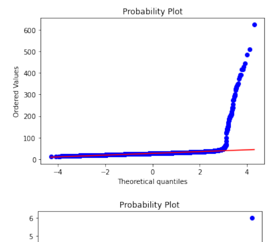
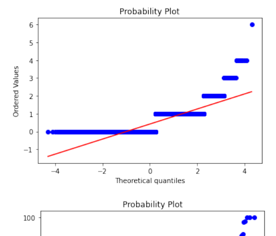
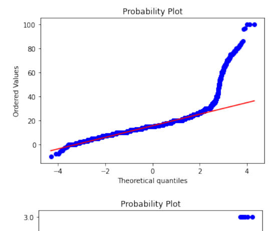
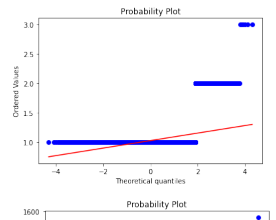
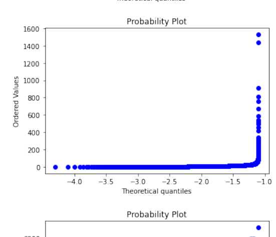
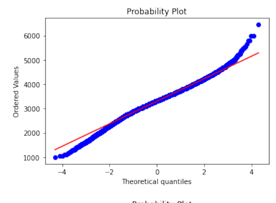
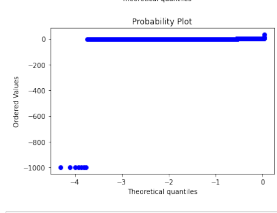

In [ ]:
