# Supplementary material for: Perinatal maternal characteristics predict a high risk of neonatal asphyxia: A multi-center retrospective cohort study in China
Source: Front Med (Lausanne). 2022 Aug 8;9:944272. doi: 10.3389/fmed.2022.944272 (PMC9393324; doi:10.3389/fmed.2022.944272)
Supplement: Supplementary Data Sheet 2 — Coding of logistic regression. [file Data_Sheet_2.pdf]

Table of Contents

1. Logistic Regression on resampled Data
   A. Model Results
   B. Performance on resampled data
   C. Performance on original data
2. Odds Ratio and Confidance Interval
3. Reference

In [1]:
```python
import pandas as pd
from scipy import stats
from scipy.stats import fisher_exact
from scipy.stats import pearsonr
import pylab
import numpy as np

from sklearn.linear_model import LogisticRegression
from sklearn.model_selection import train_test_split
import statsmodels.api as sm
from sklearn import metrics
from sklearn.metrics import roc_auc_score,confusion_matrix,accuracy_score
from statsmodels.stats.outliers_influence import variance_inflation_factor
import matplotlib.pyplot as plt

import xgboost as xgb
import imblearn
from collections import Counter
from scipy.stats import pearsonr
pd.set_option('display.max_rows', 200)
```

In [2]:
```python
d0 = pd.read_csv('/Users/manzah/Code/yuyi/d0.xlsx',low_memory=False)
d1 = pd.read_csv('/Users/manzah/Code/yuyi/d1.xlsx',low_memory=False)
```

In [3]:
```python
# create target variable
d0['target'] = 0
d1['target'] = 1
```

In [4]:
```python
df = pd.concat([d0, d1])[['H3年龄','孕周','bmi','既往分娩次数','H13增重', 'H9胎数', 'F3_4_1破膜至分娩时间小时',
                          'F9_3新生儿1-体重','F2_1_0_4第二产程时间','F3_1妊娠期高血压1是', 'F3_4胎膜早破 1是',
                          'F3_5胎儿窘迫1是','F3_6脐带缠绕1是','F3_7脐带脱垂1是','F3_10产后出血1是','F4_1胎盘形态异常1是',
                          'F4_2前置胎盘1是', 'F4_4胎盘早剥1是', 'F4_6子宫破裂1是', 'F6_3合并症-心脏病1是',
                          'F6_4合并症-肝病1是', 'F6_8合并症-自身免疫病1是', 'F9_9新生儿1-复苏1是',
                          'F9_10新生儿1-产房气管插管1是', 'F6_2_1合并症-贫血', 'F9_6新生儿1-死胎/死亡','H8受孕方式 1自然2辅助生育
',
                          'F2_1分娩方式1VD2CS','F3_5_1胎儿窘迫1胎心型2羊水型','F9_1新生儿1-性别1男2女',
                          'F9_8新生儿1-结局 1出院2转儿科3死亡4放弃','F6_5_1合并症-糖尿病','F9_8_1新生儿1-转儿科','F7_1阴道分娩操
作',
                          'F2_1_1CS指征','阴道分娩','target']]
```

In [5]:
```python
#df['年龄'] = ['[45,)' if ele  >= 45 else '[40,45)' if ele >=40 else '[35,40)' if ele >= 35 else '35-' for ele in df['H
3年龄']]
df['年龄'] = ['[40,)' if ele  >= 40 else '(,40)' for ele in df['H3年龄']]
df['孕周3741'] = [ 1 if ele  > 41  else 1 if ele <37 else 0 for ele in df['孕周']]
df['阴道分娩'] = df['阴道分娩'].fillna('不适用')
df['既往分娩'] = [1 if ele  >= 1  else 0 for ele in df['既往分娩次数']]
#df['破膜至分娩时间小时'] = ['>48' if ele  > 48  else '24-48' if ele >24 else '12-24' if ele > 12 else '0-12' if ele>=0 e
lse '无' for ele in df['F3_4_1破膜至分娩时间小时']]
df['破膜至分娩时间小时'] = ['[48,)' if ele  > 48  else '(0,48]' if ele>=0 else '无' for ele in df['F3_4_1破膜至分娩时间小时'
]]
#df['第二产程时间'] = ['>4' if ele  > 4  else '2-4' if ele >2 else '0-2' if ele>=0 else '无' for ele in df['F2_1_0_4第二
产程时间']]
df['第二产程时间'] = ['(2,)' if ele  > 2  else '(0,2)' if ele >= 0 else '无' for ele in df['F2_1_0_4第二产程时间']]
df['新生儿1体重'] = ['>4000' if ele  > 4000  else '2500-4000' if ele >2500 else '<2500' for ele in df['F9_3新生儿1-体重']
]
#df['新生儿体重254'] = [1 if ele  > 4000  else 1 if ele < 2500 else 0 for ele in df['F9_3新生儿1-体重']]
```

In [6]:
```python
#df = df.drop(columns = ['H3年龄', '孕周','F3_4_1破膜至分娩时间小时', 'F2_1_0_4第二产程时间','F9_3新生儿1-体重'] )
df = df[['既往分娩', 'H13增重', 'F3_1妊娠期高血压1是', 'F3_5胎儿窘迫1是', 'F3_6脐带缠绕1是','F4_2前置胎盘1是', 'F6_2_1合并症-贫血
',
         'H8受孕方式 1自然2辅助生育', 'F6_5_1合并症-糖尿病', '阴道分娩',
         '孕周3741','年龄', '破膜至分娩时间小时','第二产程时间', '新生儿1体重','target']]

df.rename(columns={'F6_5_1合并症-糖尿病': 'F6_5_1合并症_糖尿病',
                   'H8受孕方式 1自然2辅助生育':'H8受孕方式_1自然2辅助生育','F6_2_1合并症-贫血': 'F6_2_1合并症_贫血'}, inplace=Tr
ue)
```

In [7]:
```python
X = df.drop(columns ='target' )
y = df['target']
#df_d = pd.get_dummies(X)
```

In [ ]:
```python
for var in X.columns:
    print(var)
    print(X[var].unique())
```

In [8]:
```python
pd.DataFrame({ 'Target Class':pd.value_counts(y, sort=True,normalize=True).index,
               'Count': pd.value_counts(y, sort=True).values,
               'Pct':pd.value_counts(y, sort=True,normalize=True).values})
```

Out[8]:

	Target Class	Count	Pct
0	0	82160	0.986386
1	1	1134	0.013614

Resampling

In [9]:
```python
from imblearn.over_sampling import SMOTE
from imblearn.under_sampling import RandomUnderSampler
from imblearn.over_sampling import RandomOverSampler
from imblearn.pipeline import Pipeline
from imblearn.over_sampling import SMOTENC
```

In [10]:
```python
# define pipeline
over = RandomOverSampler(sampling_strategy=0.1,random_state=1823)
under = RandomUnderSampler(sampling_strategy=0.5,random_state=1823)
steps = [('o', over), ('u', under)]
pipeline = Pipeline(steps=steps)
# transform the dataset
X_re, y_re = pipeline.fit_resample(X, y)

#
#under = RandomUnderSampler(sampling_strategy=0.1,random_state=1823)
#X_rel, y_rel = under.fit_resample(X, y)
#smote_nc = SMOTENC(categorical_features=[0, 2, 3, 4, 5, 6, 7, 8, 9, 10, 11, 12, 13, 14],random_state=1823,sampling_st
rategy=0.5)
#X_re, y_re = smote_nc.fit_resample(X_rel, y_rel)
```

In [11]:
```python
pd.DataFrame({ 'Target Class':pd.value_counts(y_re, sort=True,normalize=True).index,
               'Count': pd.value_counts(y_re, sort=True).values,
               'Pct':pd.value_counts(y_re, sort=True,normalize=True).values})
```

Out[11]:

	Target Class	Count	Pct
0	0	16432	0.666667
1	1	8216	0.333333

Logistic Regression on resampled data

In [12]:
```python
X_re['intercept'] = 1
X_train, X_test, y_train, y_test = train_test_split(X_re, y_re, test_size=0.2, random_state=107)

tr = X_train
tr['target'] = y_train

test = X_test
test['target'] = y_test
```

Model results

In [13]:
```python
import statsmodels.formula.api as smf #, Treatment(reference=胎吸/产钳)
lm = smf.logit(formula = 'target ~   既往分娩 + F3_1妊娠期高血压1是 + F3_5胎儿窘迫1是 + \
                          F3_6脐带缠绕1是 + F4_2前置胎盘1是  +\
                          孕周3741 + C(年龄) + C(破膜至分娩时间小时) + C(第二产程时间) + C(新生儿1体重)', data = tr).fit()

#C(F6_5_1合并症_糖尿病) + F6_2_1合并症_贫血+ +  C(阴道分娩)  + H8受孕方式_1自然2辅助生育'
lm.summary()
```

```
Optimization terminated successfully.
         Current function value: 0.553073
         Iterations 5
```

Out[13]:

Logit Regression Results

Dep. Variable:	target	No. Observations:	19718
Model:	Logit	Df Residuals:	19704
Method:	MLE	Df Model:	13
Date:	Sat, 26 Mar 2022	Pseudo R-squ.:	0.1306
Time:	18:48:57	Log-Likelihood:	-10906.
converged:	True	LL-Null:	-12543.
Covariance Type:	nonrobust	LLR p-value:	0.000

	coef	std err	z	P>|z|	[0.025	0.975]
Intercept	-1.3457	0.051	-26.597	0.000	-1.445	-1.247
C(年龄)[T.[40,)]	0.1477	0.087	1.697	0.090	-0.023	0.318
C(破膜至分娩时间小时)[T.[48,)]	0.2704	0.152	1.776	0.076	-0.028	0.569
C(破膜至分娩时间小时)[T.无]	-0.1790	0.048	-3.749	0.000	-0.273	-0.085
C(第二产程时间)[T.(2,)]	0.3451	0.068	5.072	0.000	0.212	0.478
C(第二产程时间)[T.无]	0.0739	0.037	1.975	0.048	0.001	0.147
C(新生儿1体重)[T.<2500]	1.1004	0.060	18.345	0.000	0.983	1.218
C(新生儿1体重)[T.>4000]	0.3965	0.068	5.817	0.000	0.263	0.530
既往分娩	-0.1291	0.036	-3.563	0.000	-0.200	-0.058
F3_1妊娠期高血压1是	0.8702	0.067	12.944	0.000	0.738	1.002
F3_5胎儿窘迫1是	1.5591	0.047	33.142	0.000	1.467	1.651
F3_6脐带缠绕1是	0.5201	0.037	13.999	0.000	0.447	0.593
F4_2前置胎盘1是	1.0391	0.081	12.763	0.000	0.880	1.199
孕周3741	0.7758	0.046	16.878	0.000	0.686	0.866

Performance

In [277]:
```python
# performing predictions on the test datdaset
preds = lm.predict(test)
prediction = list(map(round, preds))

preds_tr = lm.predict(tr)

prediction_tr= list(map(round, preds_tr))
```

Test

In [278]:
```python
# confusion matrix
cm = confusion_matrix(y_test, prediction)
tn, fp, fn, tp = confusion_matrix(y_test, prediction).ravel()
specificity = tn / (tn+fp)
sensitivity = tp / (tp+fn)
print ("Confusion Matrix : \n", cm)

# accuracy score of the model
print('Test accuracy = ', accuracy_score(y_test, prediction))
print('Test specificity = ', round(specificity,3))
print('Test sensitivity = ',round(sensitivity,3))
print('Test AUC = ', round(roc_auc_score(y_test, prediction),3))
```

```
Confusion Matrix :
 [[2883  393]
 [ 955  699]]
Test accuracy =  0.7265720081135902
Test specificity =  0.88
Test sensitivity =  0.423
Test AUC =  0.651
```

Training

In [279]:
```python
# confusion matrix
cm = confusion_matrix(y_train, prediction_tr)
tn, fp, fn, tp = confusion_matrix(y_train, prediction_tr).ravel()
specificity = tn / (tn+fp)
sensitivity = tp / (tp+fn)
print ("Confusion Matrix : \n", cm)

# accuracy score of the model
print('Training accuracy = ', accuracy_score(y_train, prediction_tr))
print('Training specificity = ', round(specificity,3))
print('Training sensitivity = ',round(sensitivity,3))
print('Training AUC = ', round(roc_auc_score(y_train, prediction_tr),3))
```

```
Confusion Matrix :
 [[11623  1533]
 [ 3783  2779]]
Training accuracy =  0.7303986205497515
Training specificity =  0.883
Training sensitivity =  0.423
Training AUC =  0.653
```

ROC Curve

In [280]:
```python
plt.figure(dpi=1200)

fpr, tpr, thresholds = metrics.roc_curve(y_train,  preds_tr)
auc = metrics.roc_auc_score(y_train, preds_tr)
plt.plot(fpr,tpr,label='Training ROC Curve')

fpr1, tpr1, thresholds = metrics.roc_curve(y_test,  preds)
auc = metrics.roc_auc_score(y_test, preds)
plt.plot(fpr1,tpr1,label="Test ROC Curve")


plt.legend(loc=4)
plt.xlabel("False Positive Rate")
plt.ylabel("True Positive Rate")
plt.xlim([0.0, 1.0])
plt.ylim([0.0, 1.05])
plt.plot([0, 1], [0, 1], "k--", lw=2)
plt.show()

#plt.savefig('filename.pdf')
```

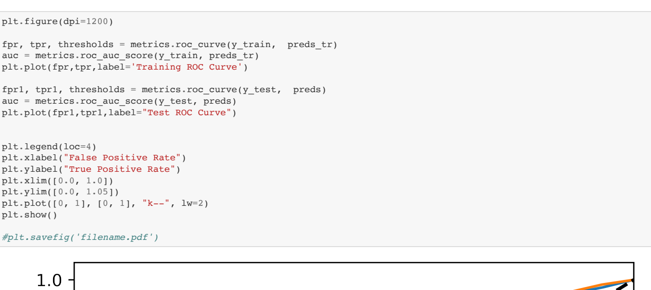

Perforamnce on original data

In [281]:
```python
X_train_o, X_test_o, y_train_o, y_test_o = train_test_split(X, y, test_size=0.2, random_state=107)
```

In [282]:
```python
# performing predictions on the test datdaset
preds = lm.predict(X_test_o)
prediction = list(map(round, preds))

preds_tr = lm.predict(X_train_o)
prediction_tr= list(map(round, preds_tr))
```

Test

In [283]:
```python
# confusion matrix
cm = confusion_matrix(y_test_o, prediction)
tn, fp, fn, tp = confusion_matrix(y_test_o, prediction).ravel()
specificity = tn / (tn+fp)
sensitivity = tp / (tp+fn)
print ("Confusion Matrix : \n", cm)

# accuracy score of the model
print('Test accuracy = ', accuracy_score(y_test_o, prediction))
print('Test specificity = ', round(specificity,3))
print('Test sensitivity = ',round(sensitivity,3))
print('Test AUC = ', round(roc_auc_score(y_test_o, prediction),3))
```

```
Confusion Matrix :
 [[14475  1932]
 [  140   112]]
Test accuracy =  0.8756227864817816
Test specificity =  0.882
Test sensitivity =  0.444
Test AUC =  0.663
```

Training

In [284]:
```python
# confusion matrix
cm = confusion_matrix(y_train_o, prediction_tr)
tn, fp, fn, tp = confusion_matrix(y_train_o, prediction_tr).ravel()
specificity = tn / (tn+fp)
sensitivity = tp / (tp+fn)
print ("Confusion Matrix : \n", cm)

# accuracy score of the model
print('Training accuracy = ', accuracy_score(y_train_o, prediction_tr))
print('Training specificity = ', round(specificity,3))
print('Training sensitivity = ',round(sensitivity,3))
print('Training AUC = ', round(roc_auc_score(y_train_o, prediction_tr),3))
```

```
Confusion Matrix :
 [[57953  7800]
 [  522   360]]
Training accuracy =  0.8751106775718466
Training specificity =  0.881
Training sensitivity =  0.408
Training AUC =  0.645
```

ROC Curve

In [285]:
```python
plt.figure(dpi=1200)

fpr, tpr, thresholds = metrics.roc_curve(y_train_o,  preds_tr)
auc = metrics.roc_auc_score(y_train_o, preds_tr)
plt.plot(fpr,tpr,label='Training ROC Curve')

fpr1, tpr1, thresholds = metrics.roc_curve(y_test_o,  preds)
auc = metrics.roc_auc_score(y_test_o, preds)
plt.plot(fpr1,tpr1,label="Test ROC Curve")


plt.legend(loc=4)
plt.xlabel("False Positive Rate")
plt.ylabel("True Positive Rate")
plt.xlim([0.0, 1.0])
plt.ylim([0.0, 1.05])
plt.plot([0, 1], [0, 1], "k--", lw=2)
plt.show()

#plt.savefig('filename.pdf')
```

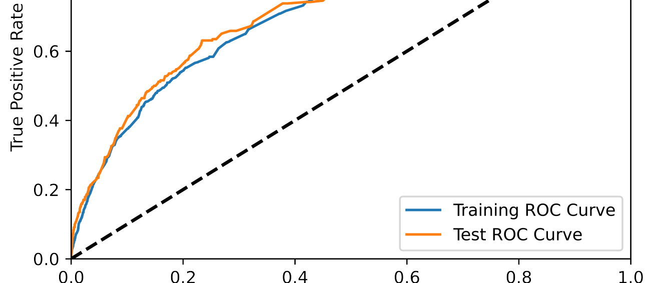

OR and CI

- **Odds Ratio in model**

In [14]:
```python
np.exp(lm.params)
```

Out[14]:
```
Intercept                    0.260364
C(年龄)[T.[40,)]              1.159112
C(破膜至分娩时间小时)[T.[48,)]     1.310529
C(破膜至分娩时间小时)[T.无]         0.836065
C(第二产程时间)[T.(2,)]         1.412073
C(第二产程时间)[T.无]           1.076698
C(新生儿1体重)[T.<2500]        3.005264
C(新生儿1体重)[T.>4000]        1.486620
既往分娩                        0.878846
F3_1妊娠期高血压1是                2.387297
F3_5胎儿窘迫1是                 4.754325
F3_6脐带缠绕1是                 1.682194
F4_2前置胎盘1是                 2.826623
孕周3741                      2.172335
dtype: float64
```

- **Confidence Interval for odds ratio**

In [17]:
```python
np.exp(lm.conf_int(alpha=0.05, cols=None))
```

Out[17]:

	0	1
Intercept	0.235784	0.287507
C(年龄)[T.[40,)]	0.977347	1.374682
C(破膜至分娩时间小时)[T.[48,)]	0.972337	1.766348
C(破膜至分娩时间小时)[T.无]	0.761351	0.918112
C(第二产程时间)[T.(2,)]	1.235802	1.613488
C(第二产程时间)[T.无]	1.000571	1.158616
C(新生儿1体重)[T.<2500]	2.671928	3.380186
C(新生儿1体重)[T.>4000]	1.300699	1.699115
既往分娩	0.818579	0.943549
F3_1妊娠期高血压1是	2.092584	2.723517
F3_5胎儿窘迫1是	4.335572	5.213524
F3_6脐带缠绕1是	1.564051	1.809260
F4_2前置胎盘1是	2.409727	3.315643
孕周3741	1.985182	2.377133

- **Odds Ratio - single variable**

In [318]:
```python
var = [ '既往分娩','F3_1妊娠期高血压1是','F3_5胎儿窘迫1是','F3_6脐带缠绕1是','F4_2前置胎盘1是','孕周3741']

var1 = [ '既往分娩','F3_1妊娠期高血压1是','F3_5胎儿窘迫1是','F3_6脐带缠绕1是','F4_2前置胎盘1是','孕周3741',
       '年龄>40','破膜至分娩时间小时[T.[48,)]','破膜至分娩时间小时[T.无]','第二产程时间[T.(2,)]',
        '第二产程时间[T.无]','新生儿1体重[T.<2500]','新生儿1体重[T.>4000]']
```

In [ ]:
```python
l  = []

for i in var:
    X = X_train[[i]]
    #X['intercept'] = 1
    log_reg = sm.Logit(y_train, X).fit()
    l.append(np.exp(log_reg.params[0]))

l.append(np.exp(smf.logit(formula = 'target ~  C(年龄)', data = tr).fit().params[1]))
l.append(np.exp(smf.logit(formula = 'target ~  C(破膜至分娩时间小时)', data = tr).fit().params[1]))
l.append(np.exp(smf.logit(formula = 'target ~  C(破膜至分娩时间小时)', data = tr).fit().params[2]))
l.append(np.exp(smf.logit(formula = 'target ~  C(第二产程时间)', data = tr).fit().params[1]))
l.append(np.exp(smf.logit(formula = 'target ~  C(第二产程时间)', data = tr).fit().params[2]))
l.append(np.exp(smf.logit(formula = 'target ~  C(新生儿1体重)', data = tr).fit().params[1]))
l.append(np.exp(smf.logit(formula = 'target ~  C(新生儿1体重)', data = tr).fit().params[2]))
```

In [326]:
```python
list(zip(var1,l))
```

Out[326]:
```
[('既往分娩', 0.4469768317950648),
 ('F3_1妊娠期高血压1是', 1.37093690248566),
 ('F3_5胎儿窘迫1是', 1.8477546549835706),
 ('F3_6脐带缠绕1是', 0.7001652892561985),
 ('F4_2前置胎盘1是', 1.4285714285714286),
 ('孕周3741', 1.3078930202217878),
 ('年龄>40', 1.2899665018344264),
 ('破膜至分娩时间小时[T.[48,)]', 1.6351850878576084),
 ('破膜至分娩时间小时[T.无]', 0.7229390616184275),
 ('第二产程时间[T.(2,)]', 1.3683071256973836),
 ('第二产程时间[T.无]', 1.3438217888125834),
 ('新生儿1体重[T.<2500]', 4.867557493073899),
 ('新生儿1体重[T.>4000]', 1.4683846724537062)]
```

Ref :

Imbalance Data (raw)

Logistic

In [126]:
```python
X_train, X_test, y_train, y_test = train_test_split(df_d, y, test_size=0.2, random_state=107)
```

In [98]:
```python
# Variable Selection with l1 penalty - LASSO
from sklearn.linear_model import LogisticRegression
log = LogisticRegression(penalty='l1', solver='liblinear')
log.fit(X_train, y_train)
```

Out[98]:
```
LogisticRegression(penalty='l1', solver='liblinear')
```

In [96]:
```python
np.array(X_train.columns)[log.coef_[0] == 0]
```

Out[96]:
```
array(['F6_8合并症-自身免疫病1是', 'H8受孕方式 1自然2辅助生育', '孕周_>=42', '破膜至分娩时间小时_无',
       '第二产程时间_无'], dtype=object)
```

In [ ]:
```python
X_train = X_train.drop(columns = ['F6_8合并症-自身免疫病1是', 'H8受孕方式 1自然2辅助生育','F6_5_1合并症-糖尿病_I型/II型',
                                  '破膜至分娩时间小时_无','F7_1阴道分娩操作_引产/点滴加强/手转胎头','F3_10产后出血1是',
                                  'F6_4合并症-肝病1是','F9_1新生儿1-性别1男2女','第二产程时间_无','F3_5_1胎儿窘迫1胎心型2羊水型',
                                   'F3_4胎膜早破 1是','F9_10新生儿1-产房气管插管1是'])
```

In [133]:
```python
log_reg = sm.Logit(y_train, X_train).fit()
log_reg.summary()
```

```
Optimization terminated successfully.
         Current function value: 0.046890
         Iterations 12
```

Out[133]:

Logit Regression Results

Dep. Variable:	target	No. Observations:	66635
Model:	Logit	Df Residuals:	66603
Method:	MLE	Df Model:	31
Date:	Mon, 21 Mar 2022	Pseudo R-squ.:	0.3339
Time:	15:11:12	Log-Likelihood:	-3124.5
converged:	True	LL-Null:	-4690.6
Covariance Type:	nonrobust	LLR p-value:	0.000

	coef	std err	z	P>|z|	[0.025	0.975]
bmi	-0.0178	0.010	-1.752	0.080	-0.038	0.002
既往分娩次数	0.0631	0.086	0.731	0.465	-0.106	0.232
H13增重	0.0108	0.007	1.481	0.139	-0.003	0.025
H9胎数	-2.2058	0.277	-7.968	0.000	-2.748	-1.663
F3_1妊娠期高血压1是	0.3502	0.136	2.580	0.010	0.084	0.616
F3_5胎儿窘迫1是	0.5769	0.103	5.577	0.000	0.374	0.780
F3_6脐带缠绕1是	0.4733	0.083	5.708	0.000	0.311	0.636
F3_7脐带脱垂1是	1.4492	0.526	2.755	0.006	0.418	2.480
F4_1胎盘形态异常1是	0.1873	0.119	1.576	0.115	-0.046	0.420
F4_2前置胎盘1是	0.5226	0.161	3.256	0.001	0.208	0.837
F4_4胎盘早剥1是	0.8426	0.250	3.371	0.001	0.353	1.332
F4_6子宫破裂1是	1.2328	0.593	2.078	0.038	0.070	2.395
F6_3合并症-心脏病1是	0.7849	0.324	2.422	0.015	0.150	1.420
F9_9新生儿1-复苏1是	0.5586	0.111	5.031	0.000	0.341	0.776
F6_2_1合并症-贫血	-0.2991	0.130	-2.296	0.022	-0.554	-0.044
F9_6新生儿1-死胎/死亡	-1.2856	0.216	-5.964	0.000	-1.708	-0.863
F2_1分娩方式1VD2CS	-2.2906	0.140	-16.306	0.000	-2.566	-2.015
F9_8新生儿1-结局 1出院2转儿科3死亡4放弃	1.0661	0.150	7.119	0.000	0.773	1.360
孕周_37-41	-0.1009	0.105	-0.962	0.336	-0.306	0.105
孕周_>=42	-0.7600	1.058	-0.718	0.473	-2.834	1.314
F6_5_1合并症-糖尿病_GDM	-4.0159	0.581	-6.914	0.000	-5.154	-2.878
F9_8_1新生儿1-转儿科_早产/感染/窒息/RDS/吸入性肺炎/湿肺	2.7539	0.151	18.259	0.000	2.458	3.050
F2_1_1CS指征_胎儿窘迫/难产	0.6839	0.138	4.959	0.000	0.414	0.954
阴道分娩_自然	-2.0017	0.127	-15.780	0.000	-2.250	-1.753
年龄_>=35	-0.0261	0.100	-0.261	0.794	-0.222	0.170
破膜至分娩时间小时_12-24	-0.3220	0.178	-1.810	0.070	-0.671	0.027
破膜至分娩时间小时_24-48	-0.6791	0.254	-2.674	0.007	-1.177	-0.181
破膜至分娩时间小时_>48	-0.4844	0.308	-1.572	0.116	-1.088	0.120
第二产程时间_2-4	-0.1401	0.174	-0.804	0.422	-0.482	0.202
第二产程时间_>4	-0.6904	0.453	-1.526	0.127	-1.577	0.197
新生儿1-体重_2500-4000	0.0666	0.113	0.589	0.556	-0.155	0.288
新生儿1-体重_>4000	0.5561	0.193	2.878	0.004	0.177	0.935

In [ ]:
```python
# VIF dataframe
# Variance Inflation Factor(VIF), check if any variables in the dataset have highly collinearity issue.

vif_data = pd.DataFrame()
vif_data["feature"] = X_train.columns

# calculating VIF for each feature
vif_data["VIF"] = [variance_inflation_factor(X_train.values, i)
                          for i in range(len(X_train.columns))]

print(vif_data)
```

performance

In [138]:
```python
X_test = X_test.drop(columns = ['F6_8合并症-自身免疫病1是', 'H8受孕方式 1自然2辅助生育','F6_5_1合并症-糖尿病_I型/II型',
                                '破膜至分娩时间小时_无','F7_1阴道分娩操作_引产/点滴加强/手转胎头','F3_10产后出血1是',
                                'F6_4合并症-肝病1是','F9_1新生儿1-性别1男2女','第二产程时间_无','F3_5_1胎儿窘迫1胎心型2羊水型',
                                 'F3_4胎膜早破 1是','F9_10新生儿1-产房气管插管1是'])

# performing predictions on the test datdaset
preds = log_reg.predict(X_test)
prediction = list(map(round, preds))

preds_tr = log_reg.predict(X_train)
prediction_tr= list(map(round, preds_tr))

# comparing original and predicted values of y
#print('Actual values', list(y_test.values))
#print('Predictions :', prediction)
```

In [139]:
```python
# confusion matrix
cm = confusion_matrix(y_test, prediction)
tn, fp, fn, tp = confusion_matrix(y_test, prediction).ravel()
specificity = tn / (tn+fp)
sensitivity = tp / (tp+fn)
print ("Confusion Matrix : \n", cm)

# accuracy score of the model
print('Test accuracy = ', accuracy_score(y_test, prediction))
print('Test specificity = ', round(specificity,3))
print('Test sensitivity = ',round(sensitivity,3))
print('Test AUC = ', round(roc_auc_score(y_test, prediction),3))
```

```
Confusion Matrix :
 [[16387    20]
 [  238    14]]
Test accuracy =  0.9845128759229246
Test specificity =  0.999
Test sensitivity =  0.056
Test AUC =  0.527
```

In [140]:
```python
# confusion matrix
cm = confusion_matrix(y_train, prediction_tr)
tn, fp, fn, tp = confusion_matrix(y_train, prediction_tr).ravel()
specificity = tn / (tn+fp)
sensitivity = tp / (tp+fn)
print ("Confusion Matrix : \n", cm)

# accuracy score of the model
print('Training accuracy = ', accuracy_score(y_train, prediction_tr))
print('Training specificity = ', round(specificity,3))
print('Training sensitivity = ',round(sensitivity,3))
print('Training AUC = ', round(roc_auc_score(y_train, prediction_tr),3))
```

```
Confusion Matrix :
 [[65692    61]
 [  837    45]]
Training accuracy =  0.986523598709387
Training specificity =  0.999
Training sensitivity =  0.051
Training AUC =  0.525
```

In [ ]:
```python
#dummy
X_tr_1 = X_train[[ 'intercept', '既往分娩', 'H13增重', 'F3_1妊娠期高血压1是', 'F3_5胎儿窘迫1是', 'F3_6脐带缠绕1是',
                  'F4_2前置胎盘1是', 'F6_2_1合并症-贫血', 'H8受孕方式 1自然2辅助生育', '孕周3741',
                  'F6_5_1合并症-糖尿病_GDM', #'F6_5_1合并症-糖尿病_I型/II型'
                   '阴道分娩_自然', #'阴道分娩_胎吸/产钳',
                  '年龄_45+', '年龄_[35,40)', '年龄_[40,35)',#'年龄_<35',
                  '破膜至分娩时间小时_0-12', '破膜至分娩时间小时_12-24', '破膜至分娩时间小时_24-48', '破膜至分娩时间小时_>48',
                  #'破膜至分娩时间小时_无',
                  '第二产程时间_0-2', '第二产程时间_2-4', '第二产程时间_>4', '第二产程时间_无',
                  '新生儿1-体重_<2500', '新生儿1-体重_>4000',
                  'F7_1阴道分娩操作_引产/点滴加强/手转胎头','F2_1_1CS指征_胎儿窘迫/难产']]
log_reg = sm.Logit(y_train, X_tr_1).fit()
log_reg.summary()


X_test = X_test[[ 'intercept', '既往分娩', 'H13增重', 'F3_1妊娠期高血压1是', 'F3_5胎儿窘迫1是', 'F3_6脐带缠绕1是',
                  'F4_2前置胎盘1是', 'F6_2_1合并症-贫血', 'H8受孕方式 1自然2辅助生育', '孕周3741',
                  'F6_5_1合并症-糖尿病_GDM', #'F6_5_1合并症-糖尿病_I型/II型',
                   '阴道分娩_自然', #'阴道分娩_胎吸/产钳',
                  '年龄_45+', '年龄_[35,40)', '年龄_[40,35)',#'年龄_<35',
                  '破膜至分娩时间小时_0-12', '破膜至分娩时间小时_12-24', '破膜至分娩时间小时_24-48', '破膜至分娩时间小时_>48',
                  #'破膜至分娩时间小时_无',
                  '第二产程时间_0-2', '第二产程时间_2-4', '第二产程时间_>4', '第二产程时间_无',
                  '新生儿1-体重_<2500', '新生儿1-体重_>4000','F7_1阴道分娩操作_引产/点滴加强/手转胎头','F2_1_1CS指征_胎儿窘迫/难
产']]
```

EDA

In [ ]:
```python
#var = ['阴道分娩','年龄', '破膜至分娩时间小时', '第二产程时间', 'H8受孕方式_1自然2辅助生育']
df[['阴道分娩','target']].groupby('阴道分娩').mean()
tr[['阴道分娩','target']].groupby('阴道分娩').mean()
df[['年龄','target']].groupby('年龄').mean()
tr[['年龄','target']].groupby('年龄').mean()
df[['破膜至分娩时间小时','target']].groupby('破膜至分娩时间小时').mean()
tr[['破膜至分娩时间小时','target']].groupby('破膜至分娩时间小时').mean()
df[['第二产程时间','target']].groupby('第二产程时间').mean()
tr[['第二产程时间','target']].groupby('第二产程时间').mean()
df[['H8受孕方式_1自然2辅助生育','target']].groupby('H8受孕方式_1自然2辅助生育').mean()
tr[['H8受孕方式_1自然2辅助生育','target']].groupby('H8受孕方式_1自然2辅助生育').mean()
```
