# Supplementary material for: Perinatal maternal characteristics predict a high risk of neonatal asphyxia: A multi-center retrospective cohort study in China
Source: Front Med (Lausanne). 2022 Aug 8;9:944272. doi: 10.3389/fmed.2022.944272 (PMC9393324; doi:10.3389/fmed.2022.944272)
Supplement: Supplementary Data Sheet 5 — Coding of exploratory data analysis. [file Data_Sheet_5.pdf]

Table of Contents

1. EDA
   A. EDA by average APGAR score
   B. EDA by percentage of severe asphyxias

In [55]:
```
import pandas as pd
from scipy import stats
from scipy.stats import fisher_exact
from scipy.stats import pearsonr
import pylab
import numpy as np

from sklearn.linear_model import LogisticRegression
from sklearn.model_selection import train_test_split
import statsmodels.api as sm
from sklearn import metrics
from sklearn.metrics import roc_auc_score,confusion_matrix,accuracy_score
from statsmodels.stats.outliers_influence import variance_inflation_factor
import matplotlib.pyplot as plt

import xgboost as xgb
import imblearn
from collections import Counter
from scipy.stats import pearsonr
pd.set_option('display.max_rows', 200)

import seaborn as sns
import matplotlib.pyplot as plt
from IPython.display import display
```

In [2]:
```
d0 = pd.read_csv('/Users/manzah/Code/yuyi/d0_1.xlsx',low_memory=False) # 82160
d1 = pd.read_csv('/Users/manzah/Code/yuyi/d1_1.xlsx',low_memory=False) #1134
```

In [3]:
```
# create target variable
#d0['target'] = 0
#d1['target'] = 1
```

In [ ]:
```
# create apgar score with min of two columns:

# 这两个数值只要一个小于等于7就诊断窒息，4-7是轻度窒息，0-3是重度窒息，非窒息组就是应该都是8-10分，
#d0['APGAR'] = pd.np.minimum(d0['F9_4_1新生儿1评分-1min'], d0['F9_4_2新生儿1-评分5min'])
d1['APGAR'] = pd.np.minimum(d1['F9_4_1新生儿1评分-1min'], d1['F9_4_2新生儿1-评分5min'])
```

abnormal values

In [6]:
```
#d0[d0['APGAR'] <= 7][['F9_4_1新生儿1评分-1min','F9_4_2新生儿1-评分5min']].shape
```

In [5]:
```
d1[d1['APGAR'] > 7][['F9_4_1新生儿1评分-1min','F9_4_2新生儿1-评分5min']].shape
```

Out[5]:
```
(49, 2)
```

remove abnormal values

In [115]:
```
#d0 = d0[d0['APGAR'] > 7]
d1 = d1[d1['APGAR'] <= 7]
```

In [116]:
```
df = d1[['H3年龄','孕周','bmi','既往分娩次数','H13增重', 'H9胎数', 'F3_4_1破膜至分娩时间小时',
                          'F9_3新生儿1-体重','F2_1_0_4第二产程时间','F3_1妊娠期高血压1是', 'F3_4胎膜早破 1是',
                          'F3_5胎儿窘迫1是','F3_6脐带缠绕1是','F3_7脐带脱垂1是','F3_10产后出血1是','F4_1胎盘形态异常1是',
                          'F4_2前置胎盘1是', 'F4_4胎盘早剥1是', 'F4_6子宫破裂1是', 'F6_3合并症-心脏病1是',
                          'F6_4合并症-肝病1是', 'F6_8合并症-自身免疫病1是', 'F9_9新生儿1-复苏1是',
                          'F9_10新生儿1-产房气管插管1是', 'F6_2_1合并症-贫血', 'F9_6新生儿1-死胎/死亡','H8受孕方式 1自然2辅助生育
',
                          'F2_1分娩方式1VD2CS','F3_5_1胎儿窘迫1胎心型2羊水型','F9_1新生儿1-性别1男2女',
                          'F9_8新生儿1-结局 1出院2转儿科3死亡4放弃','F6_5_1合并症-糖尿病','F9_8_1新生儿1-转儿科','F7_1阴道分娩操
作',
                          'F2_1_1CS指征','阴道分娩','APGAR']]
```

In [ ]:
```
#df['年龄'] = ['[45,)' if ele  >= 45 else '[40,45)' if ele >=40 else '[35,40)' if ele >= 35 else '35-' for ele in df['H
3年龄']]
df['年龄'] = ['[40,)' if ele  >= 40 else '(,40)' for ele in df['H3年龄']]
df['孕周3741'] = [ 1 if ele  > 41  else 1 if ele <37 else 0 for ele in df['孕周']]
df['阴道分娩'] = df['阴道分娩'].fillna('不适用')
df['既往分娩'] = [1 if ele  >= 1  else 0 for ele in df['既往分娩次数']]
#df['破膜至分娩时间小时'] = ['>48' if ele  > 48  else '24-48' if ele >24 else '12-24' if ele > 12 else '0-12' if ele>=0 e
lse '无' for ele in df['F3_4_1破膜至分娩时间小时']]
df['破膜至分娩时间小时'] = ['[48,)' if ele  > 48  else '(0,48]' if ele>=0 else 'None' for ele in df['F3_4_1破膜至分娩时间小
时']]
#df['第二产程时间'] = ['>4' if ele  > 4  else '2-4' if ele >2 else '0-2' if ele>=0 else '无' for ele in df['F2_1_0_4第二
产程时间']]
df['第二产程时间'] = ['(2,)' if ele  > 2  else '(0,2)' if ele >= 0 else 'None' for ele in df['F2_1_0_4第二产程时间']]
df['新生儿1体重'] = ['>4000' if ele  > 4000  else '2500-4000' if ele >2500 else '<2500' for ele in df['F9_3新生儿1-体重']
]
#df['新生儿体重254'] = [1 if ele  > 4000  else 1 if ele < 2500 else 0 for ele in df['F9_3新生儿1-体重']]
```

In [118]:
```
df = df[['既往分娩', 'H13增重', 'F3_1妊娠期高血压1是', 'F3_5胎儿窘迫1是', 'F3_6脐带缠绕1是','F4_2前置胎盘1是',
         #'F6_2_1合并症-贫血',  'H8受孕方式 1自然2辅助生育'
         'F6_5_1合并症-糖尿病', '阴道分娩',
         '孕周3741','年龄', '破膜至分娩时间小时','第二产程时间', '新生儿1体重','APGAR',
         '孕周']]

df.rename(columns={'F6_5_1合并症-糖尿病': 'F6_5_1合并症_糖尿病',
                   'H8受孕方式 1自然2辅助生育':'H8受孕方式_1自然2辅助生育','F6_2_1合并症-贫血': 'F6_2_1合并症_贫血'}, inplace=Tr
ue)
```

In [119]:
```
df.columns = ['Parity',
 'Weight-gain',
 'Hypertensive disorders',
 'Fetal distress',
 'Nuchal cord',
 'Placenta previa',
 'Gestational diabetes mellitus',
 'Mode of delivery',
 'Gestational week at birth_3741',
 'Maternal age',
 'Duration of PROM',
 'Duration of second stage of labor',
 'Birth weight',
 'APGAR',
 'Gestational week at birth']

df['Mode of delivery'] = ['Vaginal delivery' if ele  == '自然'  else 'vaginal delivery with instruments' if ele =='胎吸
/产钳' else 'N/A' for ele in df['Mode of delivery']]
```

In [120]:
```
df['severe asphyxia'] = [1 if ele  <= 3  else 0 for ele in df['APGAR']]
```

In [121]:
```
#X = df.drop(columns =['APGAR','target'] )
#y = df['target']
#df_d = pd.get_dummies(X)
```

In [ ]:
```
for var in df.columns:
    print(var)
    print(df[var].unique())
```

In [100]:
```
pd.DataFrame({ 'Target Class':pd.value_counts(y,normalize=True).index,
              'Count': pd.value_counts(y).values,
              'Pct':pd.value_counts(y,normalize=True).values})
```

Out[100]:

	Target Class	Count	Pct
0	0	987	0.910517
1	1	97	0.089483

EDA

In [149]:
```
def catEDA(df, variable, target, ylab, color = "PuBu"):
    mat = pd.DataFrame(df.groupby([variable]).mean()[target])

    # plot grouped bar chart
    chart1, ax1 = plt.subplots()
    g = sns.barplot(x=mat.index, y=mat[target], palette=color,ax=ax1)
    #g.set_xticklabels(g.get_xticklabels(), rotation=30, ha="right",fontsize=9)
    g.set(ylabel=ylab)

    plt.show()

    #Add colunm "Total obs%"
    mat["Total obs%"] = pd.value_counts(df[variable].fillna('missing').values, dropna=False, normalize=True)
    return g,display(mat)
```

In [144]:
```
def numEDA(data, target, variable, num_bins, ylab):

    # combain target value and variable to a panda dataframe
    df = pd.DataFrame()
    df["target"] = data[target]
    df["var"] = data[variable]
    #df.head()

    # sort df ascending
    df.sort_values(by="var",ascending=True, inplace=True)

    # Define df w/ wo missing
    df_notna = df.dropna() # df with non-missing values
    df_na =df[df['var'].isnull()] # df with only missing values

    rows = []
    number_bins=0
    for group in np.array_split(df_notna, num_bins): # divided data into num_bins(10) groups and calculate:
        min_value =  min(group["var"])
        max_value =  max(group["var"])
        total_target = len(group["target"])
        mean_target = group["target"].mean()
        #number_bins =number_bins+1

        rows.append({'Min Var Value':min_value,'Max Var Value':max_value,'TotalNumCases':total_target,'Target_Rate': m
ean_target})
    if df_na.empty:
        mat = pd.DataFrame(rows)
    else:
        rows.append({'Min Var Value':pd.NA,'Max Var Value':pd.NA,'TotalNumCases':df_na.shape[0],'Target_Rate': sum(df_
na["target"])/df_na.shape[0]})
        mat = pd.DataFrame(rows)

    #rearrange columns order
    columnsTitles = ['Min Var Value', 'Max Var Value', 'TotalNumCases',"Target_Rate"]
    mat = mat.reindex(columns=columnsTitles)


    ax=sns.catplot(x=list(range(1,len(mat.index)+1)), y="Target_Rate", kind="bar", palette="PuBu",data=mat).set(xlabel
='Bin',ylabel = ylab)
    ax.fig.suptitle(variable)
    plt.show()
    # Calculate total numbers of each column and append to the chart
    mat.loc['Total'] = [min(df["var"]),max(df["var"]),df.shape[0], df["target"].mean()]

    #convert NumCorrectPredictions and TotalNumCases from float to integer
    mat.TotalNumCases=mat.TotalNumCases.astype(int)

    return ax,display(mat)
```

EDA by average APGAR score

In [150]:
```
var = ['Parity','Hypertensive disorders','Fetal distress','Nuchal cord',
       'Placenta previa','Gestational diabetes mellitus','Mode of delivery',
       'Maternal age','Duration of PROM','Duration of second stage of labor','Birth weight']
for i in range(len(var)):
    catEDA(df,var[i],'APGAR','Avg APGAR Score')
```

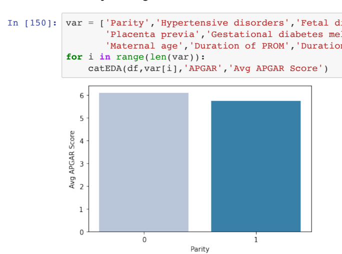

Parity	APGAR	Total obs%
0	6.109170	0.633764
1	5.732997	0.366236

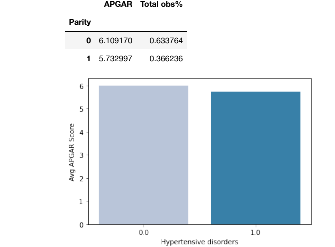

Hypertensive disorders	APGAR	Total obs%
0.0	6.000000	0.891144
1.0	5.737288	0.108856

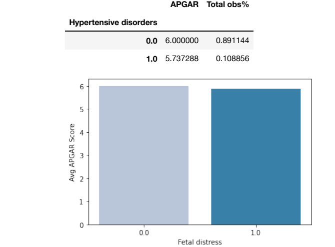

Fetal distress	APGAR	Total obs%
0.0	6.002472	0.74631
1.0	5.880000	0.25369

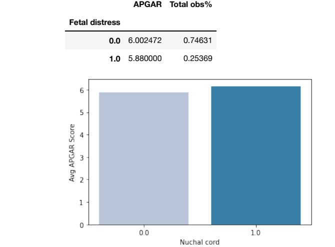

Nuchal cord	APGAR	Total obs%
0.0	5.885870	0.678967
1.0	6.152299	0.321033

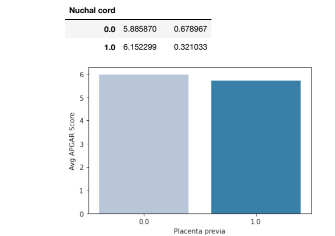

Placenta previa	APGAR	Total obs%
0.0	5.989120	0.932657
1.0	5.726027	0.067343

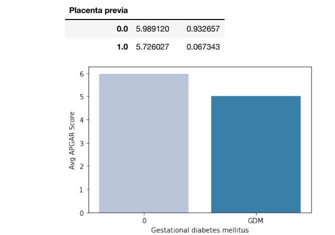

Gestational diabetes mellitus	APGAR	Total obs%
0	5.974098	0.997232
GDM	5.000000	0.002768

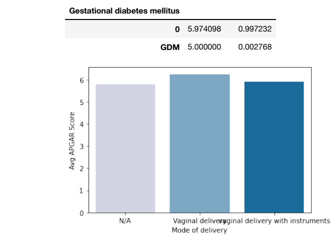

Mode of delivery	APGAR	Total obs%
N/A	5.794218	0.542435
Vaginal delivery	6.243243	0.375461
vaginal delivery with instruments	5.898876	0.082103

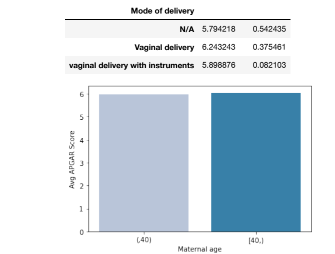

Maternal age	APGAR	Total obs%
(,40)	5.968177	0.956642
[40,)	6.042553	0.043358

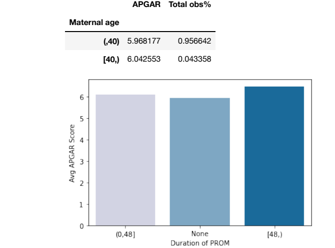

Duration of PROM	APGAR	Total obs%
(0,48]	6.087209	0.158672
None	5.939665	0.825646
[48,)	6.470588	0.015683

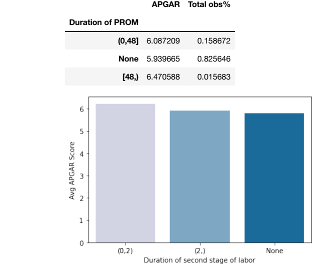

Duration of second stage of labor	APGAR	Total obs%
(0,2)	6.230216	0.384686
(2,)	5.924051	0.072878
None	5.794218	0.542435

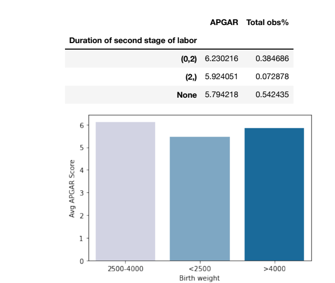

Birth weight	APGAR	Total obs%
2500-4000	6.119347	0.734317
<2500	5.474886	0.202030
>4000	5.840580	0.063653

In [151]:
```
num = ['Weight-gain','Gestational week at birth']
for i in range(len(num)):
    numEDA(df, 'APGAR', num[i], 5,'Avg APGAR Score')
```

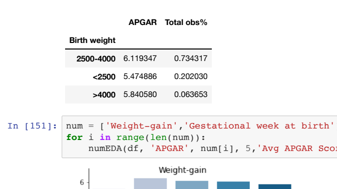

	Min Var Value	Max Var Value	TotalNumCases	Target_Rate
0	-5.00	12.00	217	5.788018
1	12.00	14.00	217	6.115207
2	14.00	15.57	217	6.027650
3	15.57	20.00	217	6.004608
4	20.00	75.00	216	5.921296
Total	-5.00	75.00	1084	5.971402

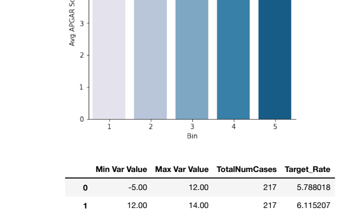

	Min Var Value	Max Var Value	TotalNumCases	Target_Rate
0	34.000000	36.000000	217	5.571429
1	36.000000	38.142857	217	5.866359
2	38.285714	39.285714	217	6.115207
3	39.285714	40.142857	217	6.013825
4	40.142857	42.285714	216	6.291667
Total	34.000000	42.285714	1084	5.971402

EDA by percentage of severe asphyxias

In [152]:
```
var = ['Parity','Hypertensive disorders','Fetal distress','Nuchal cord',
       'Placenta previa','Gestational diabetes mellitus','Mode of delivery',
       'Maternal age','Duration of PROM','Duration of second stage of labor','Birth weight']
for i in range(len(var)):
    catEDA(df,var[i],'severe asphyxia','%severe asphyxia')
```

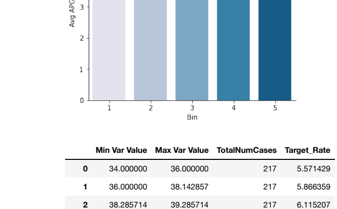

Parity	severe asphyxia	Total obs%
0	0.065502	0.633764
1	0.130982	0.366236

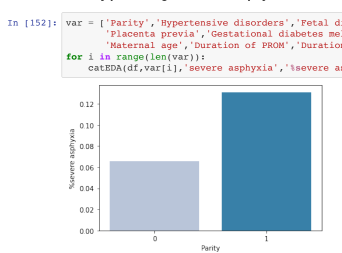

Hypertensive disorders	severe asphyxia	Total obs%
0.0	0.084886	0.891144
1.0	0.127119	0.108856

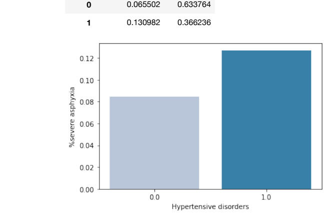

Fetal distress	severe asphyxia	Total obs%
0.0	0.087763	0.74631
1.0	0.094545	0.25369

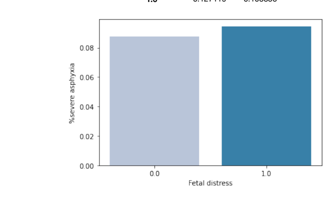

Nuchal cord	severe asphyxia	Total obs%
0.0	0.101902	0.678967
1.0	0.063218	0.321033

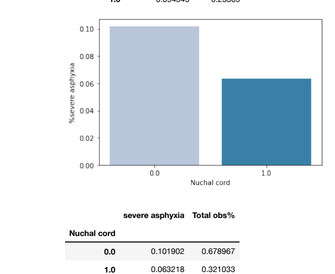

Placenta previa	severe asphyxia	Total obs%
0.0	0.088032	0.932657
1.0	0.109589	0.067343

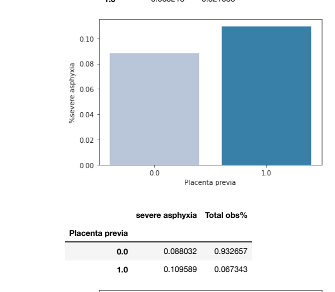

Gestational diabetes mellitus	severe asphyxia	Total obs%
0	0.089732	0.997232
GDM	0.000000	0.002768

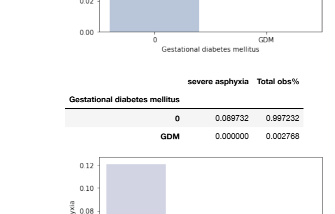

Mode of delivery	severe asphyxia	Total obs%
N/A	0.120748	0.542435
Vaginal delivery	0.049140	0.375461
vaginal delivery with instruments	0.067416	0.082103

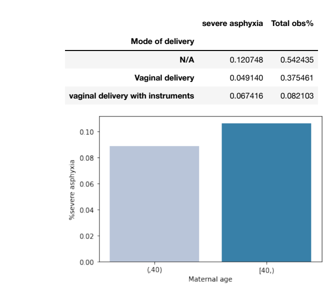

Maternal age	severe asphyxia	Total obs%
(,40)	0.088717	0.956642
[40,)	0.106383	0.043358

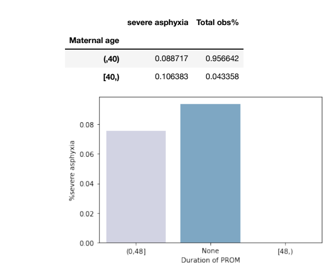

Duration of PROM	severe asphyxia	Total obs%
(0,48]	0.075581	0.158672
None	0.093855	0.825646
[48,)	0.000000	0.015683

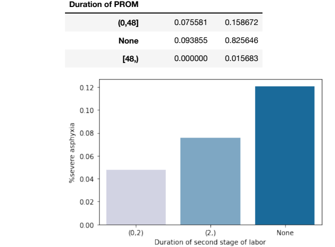

Duration of second stage of labor	severe asphyxia	Total obs%
(0,2)	0.047962	0.384686
(2,)	0.075949	0.072878
None	0.120748	0.542435

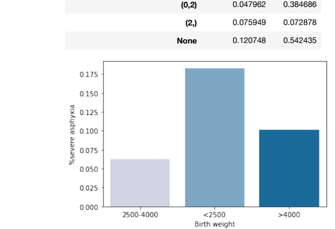

Birth weight	severe asphyxia	Total obs%
2500-4000	0.062814	0.734317
<2500	0.182648	0.202030
[illegible]	[illegible]	[illegible]

>4000	0.101449	0.063653

In [153]:
```python
num = ['Weight-gain','Gestational week at birth']
for i in range(len(num)):
    numEDA(df, 'severe asphyxia', num[i], 5,'%severe asphyxia')
```

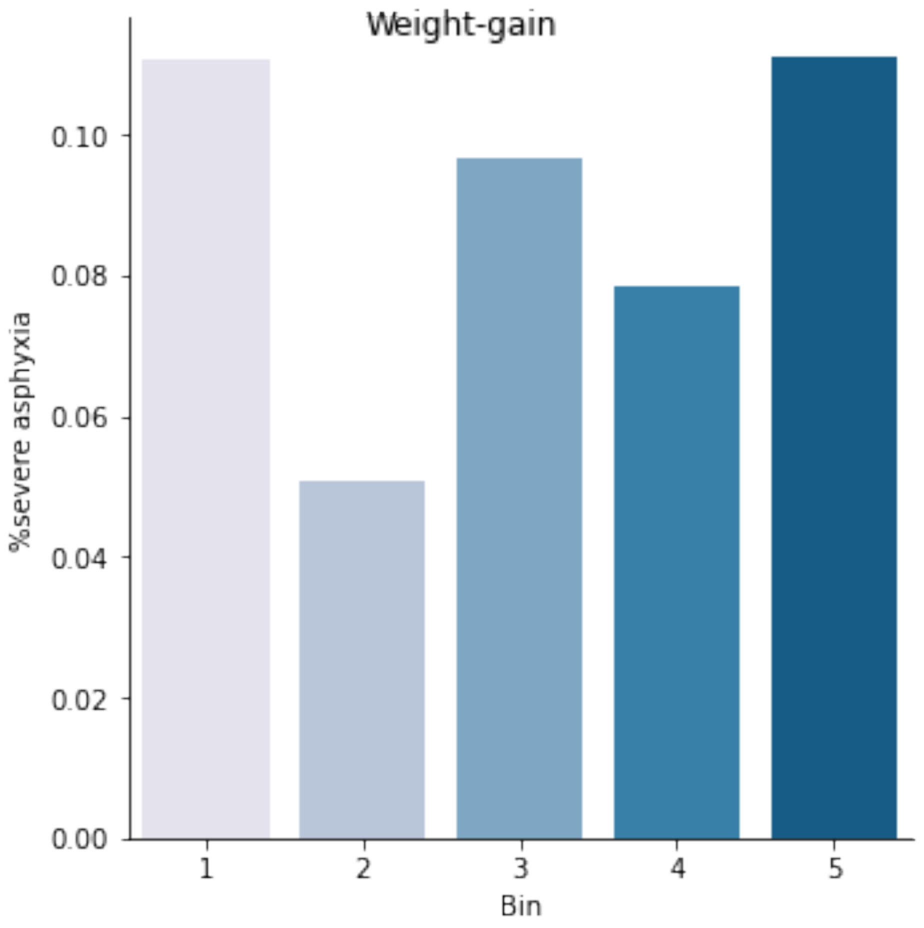

	Min Var Value	Max Var Value	TotalNumCases	Target_Rate
**0**	-5.00	12.00	217	0.110599
**1**	12.00	14.00	217	0.050691
**2**	14.00	15.57	217	0.096774
**3**	15.57	20.00	217	0.078341
**4**	20.00	75.00	216	0.111111
**Total**	-5.00	75.00	1084	0.089483

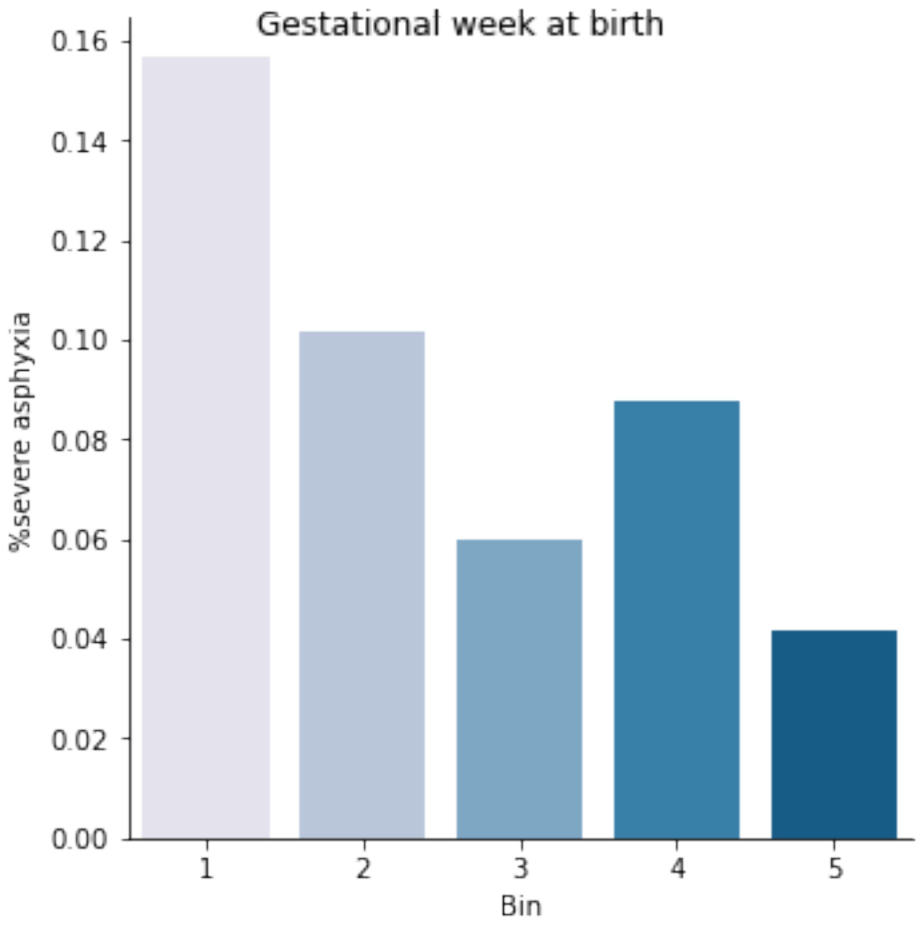

	Min Var Value	Max Var Value	TotalNumCases	Target_Rate
**0**	34.000000	36.000000	217	0.156682
**1**	36.000000	38.142857	217	0.101382
**2**	38.285714	39.285714	217	0.059908
**3**	39.285714	40.142857	217	0.087558
**4**	40.142857	42.285714	216	0.041667
**Total**	34.000000	42.285714	1084	0.089483

In [ ]:
